# Adaptive Expression and ncRNA Regulation of Genes Related to Digestion and Metabolism in Stomach of Red Pandas during Suckling and Adult Periods

**DOI:** 10.3390/ani14121795

**Published:** 2024-06-15

**Authors:** Lu Li, Liang Zhang, Lijun Luo, Fujun Shen, Yanni Zhao, Honglin Wu, Yan Huang, Rong Hou, Bisong Yue, Xiuyue Zhang

**Affiliations:** 1 Key Laboratory of Bio-Resources and Eco-Environment, Ministry of Education, College of Life Sciences, Sichuan University, Chengdu 610065, China; lilulu623@163.com (L.L.); luolijun19970120@163.com (L.L.); zhaoyanni_69@163.com (Y.Z.); bsyue@scu.edu.cn (B.Y.); 2 Integrative Science Center of Germplasm Creation in Western China (CHONGQING) Science City, Key Laboratory of Freshwater Fish Reproduction and Development (Ministry of Education), Key Laboratory of Aquatic Science of Chongqing, School of Life Sciences, Southwest University, Chongqing 400715, China; 3 The Sichuan Key Laboratory for Conservation Biology of Endangered Wildlife, Chengdu Research Base of Giant Panda Breeding, Chengdu 610081, China; lianne@panda.org.cn (L.Z.); shenfj@panda.org.cn (F.S.); hourong2000@panda.org.cn (R.H.); 4 China Conservation and Research Center for the Giant Panda, Dujiangyan 611800, China; wolong_whl@163.com (H.W.); pandayard@hotmail.com (Y.H.)

**Keywords:** red panda, ferret, digestion and metabolism, gene expression adaptation, ncRNA regulation

## Abstract

**Simple Summary:**

Red pandas have evolved to become specialized bamboo eaters within Carnivora. Probably due to the difficulty in obtaining materials, reports on genes related to digestion and metabolism at expression and regulation levels in red pandas are rare. Red pandas and carnivorous mammal ferrets have a close phylogenetic relationship. They both consume highly nutritious milk during the suckling period, but consume low-energy bamboo and high-energy meat during the adult period, respectively. In order to explore the molecular mechanisms of dietary changes and nutrient utilization in red pandas, we discussed (1) the differences in expression changes of some genes related to digestion and metabolism in the stomach of these two species after food changes, as well as the expression adaptation of genes related to digestion and metabolism in both species during different feeding periods, and (2) the regulatory effects of lncRNAs and miRNAs on adaptive expressions of genes related to digestion and metabolism in both species from suckling to adult.

**Abstract:**

Red pandas evolved from carnivores to herbivores and are unique within Carnivora. Red pandas and carnivorous mammals consume milk during the suckling period, while they consume bamboo and meat during the adult period, respectively. Red pandas and carnivorous mammal ferrets have a close phylogenetic relationship. To further investigate the molecular mechanisms of dietary changes and nutrient utilization in red pandas from suckling to adult, comparative analysis of the whole transcriptome was performed on stomach tissues from red pandas and ferrets during the suckling and adult periods. The main results are as follows: (1) we identified ncRNAs for the first time in stomach tissues of both species, and found significant expression changes of 109 lncRNAs and 106 miRNAs in red pandas and 756 lncRNAs and 109 miRNAs in ferrets between the two periods; (2) up-regulated genes related to amino acid transport regulated by lncRNA-miRNA-mRNA networks may efficiently utilize limited bamboo amino acids in adult red pandas, while up-regulated genes related to amino acid degradation regulated by lncRNAs may maintain the balance of amino acid metabolism due to larger daily intakes in adult ferrets; and (3) some up-regulated genes related to lipid digestion may contribute to the utilization of rich nutrients in milk for the rapid growth and development of suckling red pandas, while up-regulated genes associated with linoleic acid metabolism regulated by lncRNA-miRNA-mRNA networks may promote cholesterol decomposition to reduce health risks for carnivorous adult ferrets. Collectively, our study offers evidence of gene expression adaptation and ncRNA regulation in response to specific dietary changes and nutrient utilization in red pandas during suckling and adult periods.

## 1. Introduction

Diet not only sustains animal life, but also plays a pivotal role in animal evolution [1,2,3]. Mammals undergo a dietary shift from birth to adulthood: they consume breast milk during the suckling period, while their dietary habits become diverse in adulthood, including carnivorous, omnivorous, and herbivorous diets. In order to meet nutrient utilization and energy needs for mammals at different stages of growth and development, the functions of digestive organs and expression levels of genes related to digestion and metabolism must adaptively change [4,5,6,7,8,9].

After birth, red pandas begin to consume milk in order to ensure their survival, rapid growth, and development. Subsequently, they develop a complete set of teeth by approximately 6 months of age [10]. Their functional weaning, nutritional weaning, and social weaning occur at around 20 weeks, 30 weeks, and 8–11 months of age, respectively [11]. Following this period, they primarily subsist on a bamboo-based diet until the end of their lives. Red pandas typically reach adult size and mass at around 12 months of age, with sexual maturation occurring between 18 and 20 months of age [12]. The transition from carnivorous behavior to bamboo consumption in red pandas (*Ailurus fulgens*) and giant pandas (*Ailuropoda melanoleuca*), both belonging to Carnivora, has been an intriguing scientific inquiry for biologists seeking to understand how these species have adapted to consuming bamboo. Both pandas consume high-nutrient milk during the suckling period, while they exclusively consume low-nutrient bamboo during the adult period. Their morphology, genome, gut microbiota, and gene expression patterns exhibit many convergent evolutionary characteristics [13,14,15,16,17,18,19]. Research on giant pandas showed that the high expression levels of genes related to cholesterol metabolism and protein digestion and absorption in their early stages of development may help meet their high energy needs for rapid growth and development, while the high expression levels of genes related to metabolism of carbohydrate, amino acid, and protein during the adult period may indicate their high metabolic levels [9]. Further research is necessary to determine whether similar expression changes occur in digestion- and metabolism-related genes in red pandas from their infancy to adulthood.

The regulation of gene expression is a complex process. Phenotypic changes that cannot be easily explained by the genome may be attributed to epigenetic modifications, such as DNA methylation, non-coding RNAs (ncRNAs), and histone modification, without altering the DNA sequences [20]. Organisms contain a large number of ncRNAs, and while the functions of most ncRNAs remain unclear, previous studies have demonstrated their significant regulatory roles in various biological processes [21,22,23]. Long non-coding RNAs (lncRNAs) and microRNAs (miRNAs) are two widely studied types of ncRNAs. LncRNAs are defined as ncRNA transcripts with a length greater than 200 nucleotides [24,25], while miRNAs are endogenous single-stranded ncRNAs approximately 18–25 nucleotides in length [26,27]. A previous study conducted on giant pandas’ livers and pancreas revealed that DNA methylation can regulate the expression levels of genes related to digestion and metabolism during different feeding periods to adapt to dietary shifts from milk to bamboo [8]. As ncRNAs exhibit strong developmental stage specificity, both lncRNAs and miRNAs play a crucial role in regulating gene expression, thereby impacting growth, development, digestion, and metabolism. They are also involved in nutritional metabolism such as glucose metabolism, lipid metabolism, and amino acid metabolism [28,29,30,31,32,33,34,35]. However, there is a scarcity of reports on the regulatory effects of lncRNAs and miRNAs on gene expression changes related to digestion and metabolism during two different feeding periods in red pandas or giant pandas.

Red pandas, which evolved from carnivores to herbivores, are unique within the Carnivora order. Both red pandas and other carnivorous mammals consume milk during the suckling period, but they switch to bamboo and meat during the adult period, respectively. It is worth noting that red pandas and mustelids within the Carnivora order have a close phylogenetic relationship [36]. Ferrets (*Mustela putorius furo*) within the mustelid family consume milk before weaning, and then transition to a diet of meat from rodents, rabbits, and poultry after weaning [37,38,39]. In contrast to low-fat and low-protein bamboo, milk and meat are both high in fat and protein content, and exhibit similar amino acid and fatty acid compositions [40,41,42,43]. Therefore, a comparative study of red pandas and ferrets was conducted to comprehensively understand the dietary pattern features of red pandas.

How do red pandas digest and utilize nutrients from different foods during the suckling period and adult bamboo-eating period to adapt to dietary changes? The expression and regulation of genes related to digestion and metabolism in these two species likely undergo changes to adapt to or effectively utilize different diets during the two feeding periods. Therefore, in order to investigate the molecular mechanisms of dietary changes and nutrient utilization at the gene expression and ncRNA regulation levels in red pandas from suckling to adulthood, the whole transcriptome analysis was conducted using mRNA, lncRNA, and miRNA data from stomach tissue samples of red pandas and ferrets during both the suckling and adult periods.

## 2. Materials and Methods

### 2.1. Sampling

Stomach tissue samples of red pandas during suckling period and adult period were provided by the Chengdu Research Base of Giant Panda Breeding and the China Conservation and Research Center for the Giant Panda. Stomach tissue samples of ferrets during suckling period and adult period were provided by Wuxi Kuboyi Biotechnology Co., Ltd (Wuxi, China). All the animal work was conducted according to the guidelines approved by the Ethics Committee, College of Life Sciences, Sichuan University (Grant No: 20190506001). All red pandas used in this study died of natural causes. All healthy ferrets were anesthetized and euthanized, and their stomach tissue samples were dissected by our laboratory. Six stomach tissue samples from suckling period (SRW1, SRW2, SRW3) and adult period (ARW1, ARW2, ARW3) of red pandas were collected; six stomach tissue samples from suckling period (SFW1, SFW2, SFW3) and adult period (AFW1, AFW2, AFW3) of ferrets were collected. There were no pathological changes in any of these stomach tissue samples. All samples were stored at −80 °C before RNA extraction.

### 2.2. RNA Extraction and Library Preparation for Sequencing

Total RNA was extracted from stomach tissue samples of red pandas and ferrets during suckling period and adult period using Trizol reagent (Invitrogen, Carlsbad, CA, USA) according to manufacturer’s instructions. Purity of RNA was checked using NanoPhotometer spectrophotometer (Implen, Westlake Village, CA, USA), and concentration was measured using Qubit RNA Assay Kit in Qubit 2.0 Flurometer (Life Technologies, Carlsbad, CA, USA). Integrity of RNA was assessed using Agilent 2100 RNA Nano 6000 Assay Kit (Agilent Technologies, Santa Clara, CA, USA).

Library preparations for lncRNAs and mRNAs of red pandas and ferrets using strand-specific RNA-seq were carried out using NEBNext^®^ UltraTM Directional RNA Library Prep Kit for Illumina^®^ (NEB, Ipswich, MA, USA) following manufacturer’s recommendations. Paired-end reads of 150 bp were generated using Illumina NovaSeq 6000 platform in Novogene (Beijing, China). NGS QC TOOLKIT [44] was performed to remove low quality paired-end reads or reads containing adaptors. Reference genome and annotation of the red panda were obtained from DNA ZOO (https://www.dnazoo.org/) accessed on 13 October 2022, and reference genome and annotation of the ferret were obtained using Ensembl release 101. After quality control, processed reads were aligned to the corresponding reference genome using HISAT2 version 2.1.0 [45]. Transcripts were assembled and annotated using StringTie version 1.3.6 [46]. Transcripts (fasta format) were then extracted from the GTF file using gffread tool of Cufflinks version 2.2.1 [47]. Putative lncRNAs were identified using the following criteria: (1) the transcripts annotated with “u, x, i, j, o” were extracted and retained using Gffcompare version 0.11.2 [48]; (2) length ≥ 200 bp and exon number ≥ 2; (3) the transcripts with known ncRNAs (lncRNA, rRNA, tRNA, miRNA, snRNA, snoRNA, scaRNA, misc_RNA, mitochondria tRNA, and mitochondria rRNA) annotated using ENSEMBL for ferrets were removed; (4) the transcripts with low expression levels (counts < 5) in all samples were discarded; (5) the transcripts with no coding ability predicted by CPC 2.0 [49] and CNCI [50] computational tools were retained; (6) the transcripts without coding potential identified by PfamScan version 1.6.2 [51] based on amino acid sequence alignment to Pfam database were retained. We considered the transcripts meeting the above criteria as novel assembled lncRNAs. And these novel assembled lncRNAs were combined with annotated lncRNAs for subsequent analyses.

Library preparations for small RNAs of red pandas and ferrets using sRNA-seq of red pandas and ferrets were carried out using NEBNext^®^ Multiplex Small RNA Library Prep Set for Illumina^®^ (NEB, Ipswich, MA, USA) following manufacturer’s recommendations. Single-end reads of 50 bp were generated using Illumina NovaSeq 6000 platform in Novogene (Beijing, China). After sequencing all reads, the high-quality reads without adaptors were considered appropriate for further analyses. Then, the high-quality reads without short repeat sequences and other sRNAs were obtained through the following filtering steps: (1) 18 bp ≤ length ≤ 30 bp; (2) the short repeat sequences identified using RepeatMasker were discarded; (3) the non-miRNAs (such as rRNA, snoRNA, snRNA, tRNA, and lncRNA) detected by BlastN [52] for comparing sequences with Rfam database [53] were removed. After removing non-miRNAs, miRDeep2 version 0.1.3 [54] was used to analyze the remaining reads: these reads were mapped to corresponding reference genomes using mapper module of mirDeep2 [55]; the sequences of miRNA precursors and mature miRNAs provided by MiRBase database [56] were used to identify conserved miRNAs and predict novel miRNAs; all expression levels of miRNAs were calculated using quantifier module of mirDeep2.

### 2.3. Differentially Expressed lncRNAs, miRNAs, mRNAs, and Enrichment Analyses

We created all comparisons of differentially expressed analyses between the suckling group and adult group of red pandas or ferrets using R “edgeR” package [57], which takes a raw read count matrix (mRNAs, lncRNAs or miRNAs) as input. Normalized expression matrices of all samples were log-transformed. Principal component analysis (PCA) was performed on these converted data using “prcomp” function in R package “stats”. Spearman’s correlation distance between samples was analyzed using “cor” function in R; then, it was visualized using “heatmap.2” in “gplots” package. Significant differentially expressed mRNAs (DE-mRNAs), differentially expressed lncRNAs (DE-lncRNAs), and differentially expressed miRNAs (DE-miRNAs) during two different feeding periods were considered to be those with Padj < 0.05 and |log2FC| > 1. All expressed genes were selected as backgrounds in functional enrichment analyses. Enrichment analyses of Gene Ontology (GO) categories and Kyoto Encyclopedia of Genes and Genomes (KEGG) pathways were performed using enrich function of R package “clusterProfiler” [58]. Significantly enriched GO categories and KEGG pathways were considered to be those with *p* < 0.05.

### 2.4. Constructions of Regulatory Networks among lncRNAs, miRNAs, and mRNAs

Based on expression matrices of mRNAs and lncRNAs, the correlation can be calculated using cor.test function with R built-in package. LncRNAs and potential target mRNAs were extracted with *p* < 0.05 and |r| > 0.95 [59]. MiRNAs and potential target mRNAs were predicted by the intersection of the results of miRanda version 3.3a [60] and RNAhybrid version 2.1.2 [61] software.

LncRNA-miRNA-mRNA networks were based on the theory that lncRNAs can act as miRNA sponges and competitively bind miRNAs to regulate mRNA expression by using shared miRNA response elements [62,63]. The expression levels of lncRNAs and mRNAs are positively correlated, while the expression levels of lncRNAs and miRNAs are negatively correlated. To better comprehend the relationships among DE-mRNAs, DE-lncRNAs, and DE-miRNAs between the suckling group and adult group in red pandas or ferrets, the lncRNAs-mediated lncRNA-miRNA-mRNA networks were constructed. MiRanda [60] and RNAhybrid [61] software were used to predict relationships between DE-lncRNAs and DE-miRNAs. Next, these two software programs were used to obtain DE-miRNAs-targeted DE-mRNAs. Finally, in both red pandas and ferrets, we constructed regulatory networks of up-regulated DE-lncRNAs, down-regulated DE-miRNAs, and up-regulated DE-mRNAs that may interact with each other; we also constructed regulatory networks of down-regulated DE-lncRNAs, up-regulated DE-miRNAs, and down-regulated DE-mRNAs that may interact with each other. In addition, Cytoscape version 3.7.1 was used for visualizing and mapping the results.

### 2.5. Real-Time Quantitative PCR

The isolated RNAs of red pandas during suckling period and adult period were converted to double-stranded cDNAs using M5 Sprint qPCR RT Kit (Mei5 Biotechnology, Beijing, China). After the cDNA synthesis, mRNA expression levels of 9 DE-mRNAs associated with digestion and metabolism of red pandas were quantified by real-time PCR performed on the CFX96 Real-Time PCR Detection System (Bio-Rad, Hercules, CA, USA).

## 3. Results

### 3.1. RNA Sequencing

To reveal the molecular mechanisms of dietary shift and nutrient utilization of red pandas during the suckling period and the adult bamboo-eating period, we prepared whole transcriptome sequencing libraries (strand-specific RNA-seq libraries and sRNA-seq libraries) on 12 stomach tissue samples of red pandas and ferrets during the suckling and adult periods. The descriptions of the 12 stomach tissue samples of the two mammals for strand-specific RNA-seq are provided in Table S1. All the strand-specific RNA-seq data generated 38 to 49 million clean paired-end reads. We aligned each of the 12 strand-specific RNA-seq libraries to their corresponding reference genome and found that the final efficiency of the read alignments ranged from 73.58 to 93.37% (Table S1). The descriptions of the 12 stomach tissue samples of the two mammals for sRNA-seq are provided in Table S2. All the sRNA-seq data generated 6 to 8 million clean single-end reads. We aligned each of the 12 sRNA-seq libraries to their corresponding reference genome and found that the final efficiency of the read alignments ranged from 84.62 to 90.49% (Table S2).

### 3.2. Identifications and General Characteristics of lncRNAs and miRNAs

**Figure 1:**
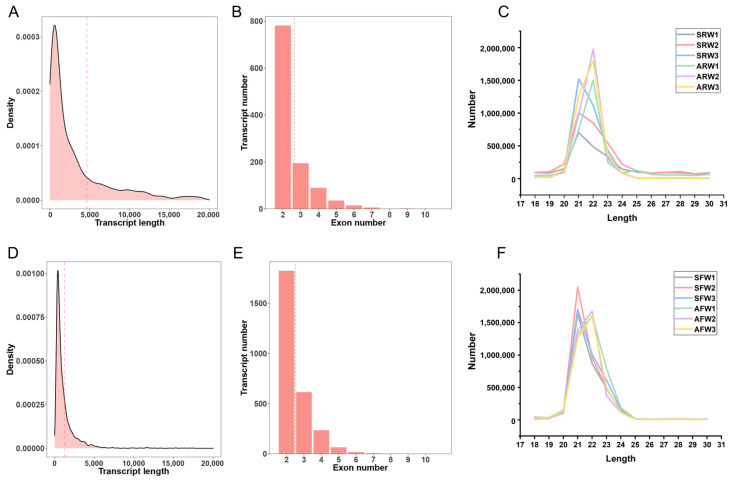
Characteristics of lncRNAs and miRNAs in red pandas and ferrets. The distribution of transcript length of lncRNAs in the stomach samples of red pandas. The dotted line shows average length (**A**). Exon number distribution of lncRNAs in the stomach samples of red pandas. The dotted line shows average number (**B**). The length distribution of miRNAs in the stomach samples of red pandas (**C**). The distribution of transcript length of lncRNAs in the stomach samples of ferrets. The dotted line shows average length (**D**). Exon number distribution of lncRNAs in the stomach samples of ferrets. The dotted line shows average number (**E**). The length distribution of miRNAs in the stomach samples of ferrets (**F**).

The known lncRNAs of red pandas are not annotated in the genome. After the assembly of the transcriptome, a series of strict filtering procedures were used to screen and identify 1134 novel lncRNA transcripts in the stomach tissue samples of red pandas (Figure S1A). In the stomach samples of red pandas, the number of novel lncRNAs annotated with “u” using Gffcompare was the largest (Figure S1B), the average length of novel lncRNAs was 4635 nt (Figure 1A), and the number of novel lncRNAs with two exons is the highest (Figure 1B). In addition to the known lncRNAs annotated in the reference genome, 474 novel lncRNA transcripts were identified in the stomach tissue samples of ferrets (Figure S1E). In the stomach samples of ferrets, the number of novel lncRNAs annotated with “u” using Gffcompare was also the largest (Figure S1F), the average length of all lncRNAs was 1246 nt (Figure 1D), and the number of all lncRNAs with two exons is the highest (Figure 1E).

In the stomach samples of red pandas, the majority of mature miRNAs were 20–24 nt in length (Figure 1C). The number of miRNAs of the suckling group and the adult group in the stomach samples of red pandas were 248 (29 novel, 219 conserved) and 235 (25 novel, 210 conserved), respectively (Figure S1C). The number of conserved miRNAs shared by the suckling group and the adult group of red pandas were 206 (Figure S1D). In the stomach samples of ferrets, the majority of mature miRNAs were also 20–24 nt in length (Figure 1F). The number of all miRNAs of the suckling group and the adult group in the stomach samples of ferrets were 254 (38 novel, 216 conserved) and 244 (30 novel, 214 conserved), respectively (Figure S1G). The number of conserved miRNAs shared by the suckling group and the adult group of ferrets were 211 (Figure S1H).

### 3.3. Sample Cluster Analyses

**Figure 2:**
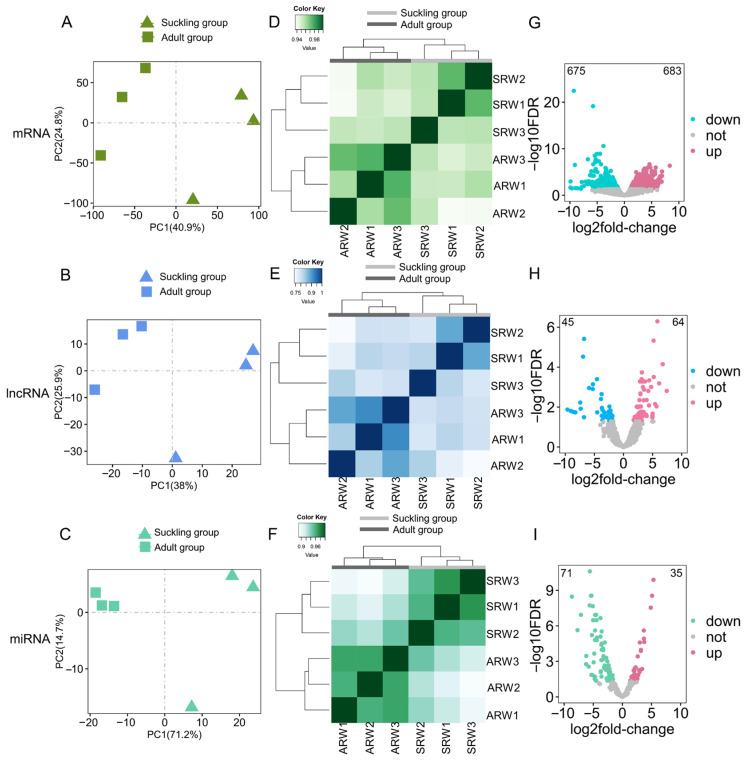
Cluster analyses for all samples and differential analyses between two feeding periods in red pandas. The expression levels of mRNAs (**A**), lncRNAs (**B**), and miRNAs (**C**) were normalized and log-transformed to perform PCA in stomach tissue samples of the suckling group and the adult group in red pandas. Different groups are represented by different shapes. The expression levels of mRNAs (**D**), lncRNAs (**E**), and miRNAs (**F**) were normalized and log-transformed to perform cluster analyses in stomach tissue samples of the suckling group and the adult group in red pandas. Distance between samples was measured using Spearman’s rank correlation coefficient. Volcano plots of mRNAs (**G**), lncRNAs (**H**), and miRNAs (**I**) in stomach samples between the suckling group and the adult group in red pandas. The number in the upper left corner and the number in the upper right corner indicate the number of down-regulated RNAs and up-regulated RNAs in adult group compared with the suckling group in red pandas, respectively. Each dot represents one RNA.

**Figure 3:**
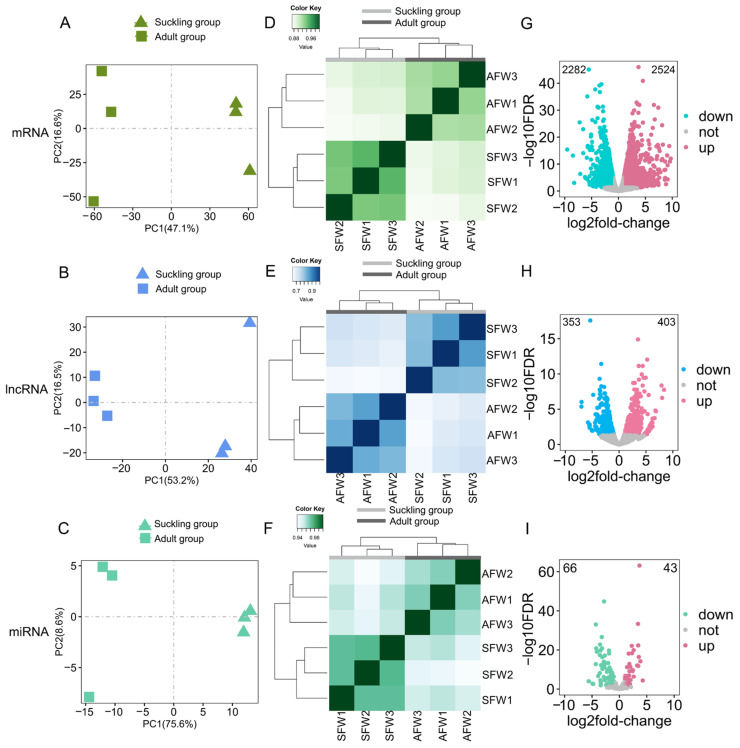
Cluster analyses for all samples and differential analyses between two feeding periods in ferrets. The expression levels of mRNAs (**A**), lncRNAs (**B**), and miRNAs (**C**) were normalized and log-transformed to perform PCA in stomach tissue samples of the suckling group and the adult group in ferrets. Different groups are represented by different shapes. The expression levels of mRNAs (**D**), lncRNAs (**E**), and miRNAs (**F**) were normalized and log-transformed to perform cluster analyses in stomach tissue samples of the suckling group and the adult group in ferrets. Distance between samples was measured using Spearman’s rank correlation coefficient. Volcano plots of mRNAs (**G**), lncRNAs (**H**), and miRNAs (**I**) in stomach samples between the suckling group and the adult group in ferrets. The number in the upper left corner and the number in the upper right corner indicate the number of down-regulated RNAs and up-regulated RNAs in adult group compared with the suckling group in ferrets, respectively. Each dot represents one RNA.

The PCA (Figure 2A–C) and hierarchical clustering analyses based on Spearman’s correlation coefficients (Figure 2D–F) of mRNAs (Figure 2A,D), lncRNAs (Figure 2B,E), and miRNAs (Figure 2C,F) in the stomach tissue samples during the suckling and adult periods of red pandas showed that the biological replicates during the same periods in red pandas were well clustered, and the samples during the different periods were well separated, indicating a significant difference in the expression patterns of mRNAs, lncRNAs, and miRNAs in stomach tissue samples during two different feeding periods. Similarly, the PCA (Figure 3A–C) and hierarchical clustering analyses (Figure 3D–F) of mRNAs (Figure 3A,D), lncRNAs (Figure 3B,E), and miRNAs (Figure 3C,F) in the stomach tissue samples during the suckling and adult periods of ferrets showed that the biological replicates during the same periods in ferrets were well clustered, and the samples during the different periods were well separated.

### 3.4. Enrichment Analyses of DE-mRNAs and Identifications of Digestion- and Metabolism-Related DE-mRNAs

**Figure 4:**
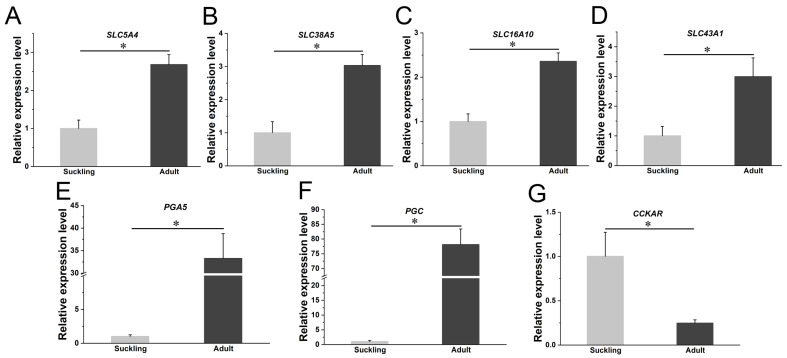
Real-time PCR of some digestion- and metabolism-related DE-mRNAs in red pandas. The relative expression levels of glucose transporter (**A**), amino acid transporters (**B**–**D**), proteases (**E**,**F**), cholecystokinin receptor (**G**)-related DE-mRNAs in stomach samples between suckling group and adult group of red pandas. The X axis represents different groups; the Y axis represents the relative mRNA expression levels; * represents significant differences in mRNA expression levels.

To explore the expression adaptation of genes related to digestion and metabolism in red pandas during the suckling period and the adult bamboo-eating period, as well as the differences in expression changes of genes related to digestion and metabolism in red pandas and ferrets after dietary changes, we analyzed the expression characteristics of DE-mRNAs between two different feeding periods in the stomach of red pandas and ferrets, respectively. An amount of 683 up- and 675 down-regulated DE-mRNAs (Figure 2G) in the adult group compared with the suckling group were identified in red pandas. The categories and pathways of the enrichment results are shown in Tables S3–S6 and Figure S2. The up-regulated DE-mRNAs of adult red pandas were involved in amino acid transport, such as amino acid transport (GO:0006865) and amino acid transmembrane transport (GO:0003333) categories. The down-regulated DE-mRNAs of adult red pandas were involved in digestion and metabolism, including response to nutrient (GO:0007584), response to fatty acid (GO:0070542), and digestive tract development (GO:0048565) categories. Compared with the suckling red pandas, we identified the up-regulated DE-mRNAs related to glucose utilization (*SLC5A4*), amino acid transport (*SLC38A5*, *SLC16A10*, *SLC43A1*), and pepsinogens (*PGA5*, *PGB*, *PGC*) in the adult group, and identified the down-regulated DE-mRNAs involved in protein digestion (*GRP*), protein and lipid digestion (*CCKAR*) in the adult group (Table S7). We subsequently validated expression levels of some DE-mRNAs related to digestion and metabolism in the stomach samples of red pandas (Figure 4). All the primer sequences used for amplification of those nine mRNAs were shown in Table S8. Similarly, 2524 up- and 2282 down-regulated DE-mRNAs (Figure 3G) in the adult group compared with the suckling group were identified in ferrets. The categories and pathways of the enrichment results are shown in Tables S9–S12 and Figure S3. The up-regulated DE-mRNAs of adult ferrets were enriched to linoleic acid metabolism (ko00591). The down-regulated DE-mRNAs of adult ferrets were enriched to digestive tract development (GO:0048565) category. Compared with the suckling ferrets, we identified the up-regulated DE-mRNAs related to linoleic acid metabolism (*ENSMPUG00000009617*, *ENSMPUG00000017129*) and amino acid degradation (*BCKDHB*, *AASS*) in adult ferrets (Table S13 and Figure S4).

### 3.5. Constructions of lncRNA-mRNA Networks

**Figure 5:**
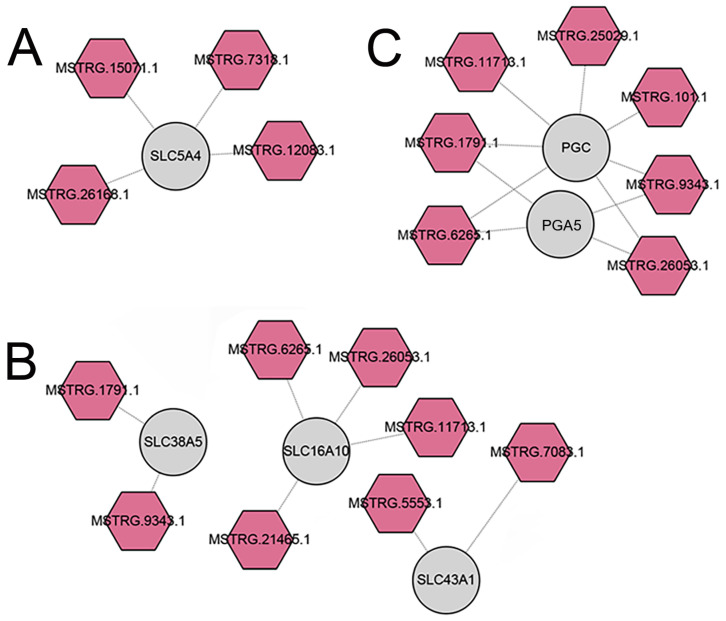
Digestion- and metabolism-related lncRNA-mRNA networks in red pandas. The interaction networks of DE-lncRNAs and DE-mRNAs associated with glucose transport (**A**), amino acid transport (**B**), and protease (**C**) in stomach samples between suckling and adult group of red pandas. The palevioletred hexagons represent the up-regulated DE-lncRNAs of adult group; the circles represent the target mRNAs of DE-lncRNAs.

To study the regulatory effects of lncRNAs on adaptive expression of digestion- and metabolism-related genes in red pandas to two different diets, we analyzed the regulatory characteristics of DE-lncRNAs between two different feeding periods on genes in the stomach of red pandas and ferrets, respectively. An amount of 64 up- and 45 down-regulated DE-lncRNAs (Figure 2H) in the adult group compared with the suckling group were identified in red pandas. The enrichment results of potential target mRNAs for DE-lncRNAs are shown in Tables S14–S17 and Figure S5. Compared with the suckling group, the enrichment of the target mRNAs of up-regulated DE-lncRNAs in the adult red pandas includes amino acid transport (GO:0006865) and peptidase activity (GO:0008233) categories. And the enrichment of the target mRNAs of down-regulated DE-lncRNAs in the adult red pandas includes regulation of protein catabolic process (GO:0042176) category. Table S18 and the interaction network showed the regulatory relationship between DE-lncRNAs and corresponding potential target DE-mRNAs during two different feeding periods related to glucose transport (Figure 5A), amino acid transport (Figure 5B), and protease (Figure 5C) in the stomach of red pandas. The involved DE-lncRNAs were up-regulated in the adult group. These DE-lncRNAs may play roles in regulating the processes of digestion, metabolism, and nutrient utilization in red pandas. Similarly, 403 up- and 353 down-regulated DE-lncRNAs (Figure 3H) in the adult group compared with the suckling group were identified in ferrets. The enrichment results of potential target mRNAs for DE-lncRNAs are shown in Tables S19–S22 and Figure S6. Compared with the suckling group, the target mRNAs of up-regulated DE-lncRNAs in adult ferrets were enriched to lipid transporter activity (GO:0005319) category, fatty acid degradation (ko00071) pathway, and valine, leucine, and isoleucine degradation (ko00280) pathway. And the target mRNAs of down-regulated DE-lncRNAs in adult ferrets were enriched to protein catabolic process (GO:0030163) category and beta-alanine metabolism (ko00410) pathway. Table S23 and the interaction network showed the regulatory relationship between DE-lncRNAs and corresponding potential target DE-mRNAs during two different feeding periods related to linoleic acid metabolism (Figure S7A) and amino acid degradation (Figure S7B) in the stomach of ferrets. The involved DE-lncRNAs were up-regulated in the adult group.

### 3.6. Constructions of miRNA-mRNA Networks

To investigate the regulatory effects of miRNAs on the adaptive expression of genes related to digestion and metabolism in red pandas from the suckling period to the adult period, we analyzed the regulatory characteristics of DE-miRNAs between two different feeding periods on genes in the stomach of red pandas and ferrets, respectively. An amount of 35 up- and 71 down-regulated DE-miRNAs (Figure 2I) in the adult group compared with the suckling group were identified in red pandas. The enrichment results of potential target mRNAs for DE-miRNAs are shown in Tables S24–S27 and Figure S8. Compared with the suckling group, the enrichment of the target mRNAs of up-regulated DE-miRNAs in the adult red pandas includes digestive system development (GO:0055123) and positive regulation of lipid transport (GO:0032370) categories. And the enrichment of the target mRNAs of down-regulated DE-miRNAs in the adult red pandas includes amino acid transport (GO:0006865) category. Similarly, 43 up- and 66 down-regulated DE-miRNAs (Figure 3I) in the adult group compared with the suckling group were identified in ferrets. The enrichment results of potential target mRNAs for DE-miRNAs are shown in Tables S28–S31 and Figure S9. Compared with the suckling group, the enrichment of the target mRNAs of up-regulated DE-miRNAs in adult ferrets includes carbohydrate binding (GO:0030246) category. And the enrichment of the target mRNAs of down-regulated DE-miRNAs in adult ferrets includes lipase activity (GO:0016298) category.

### 3.7. Constructions of lncRNA-miRNA-mRNA Networks

**Figure 6:**
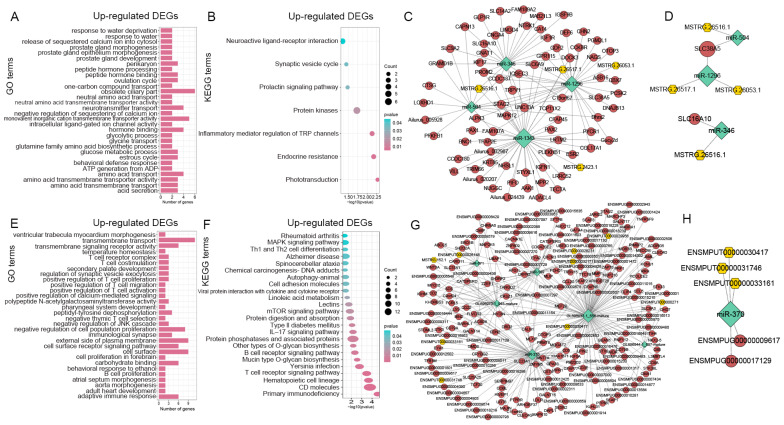
Enrichment analyses and lncRNA-miRNA-mRNA networks in red pandas and ferrets. Significantly enriched GO categories (**A**) and KEGG pathways (**B**) for DE-mRNAs of regulatory network composed of up-regulated DE-lncRNAs, down-regulated DE-miRNAs, and up-regulated DE-mRNAs in stomach samples of adult group compared with the suckling group in red pandas. The significantly enriched top 30 categories were selected for display. Regulation network of up-regulated DE-lncRNAs, down-regulated DE-miRNAs, and up-regulated DE-mRNAs that may interact in stomach samples between suckling group and adult group of red pandas (**C**). Regulation network of up-regulated DE-lncRNAs, down-regulated DE-miRNAs, and up-regulated DE-mRNAs that may interact related to amino acid transport in stomach samples between suckling group and adult group of red pandas (**D**). Significantly enriched GO categories (**E**) and KEGG pathways (**F**) for DE-mRNAs of regulatory network composed of up-regulated DE-lncRNAs, down-regulated DE-miRNAs, and up-regulated DE-mRNAs in stomach samples of adult group compared with the suckling group in ferrets. The significantly enriched top 30 categories were selected for display. Regulation network of up-regulated DE-lncRNAs, down-regulated DE-miRNAs, and up-regulated DE-mRNAs that may interact in stomach samples between suckling group and adult group of ferrets (**G**). Regulation network of up-regulated DE-lncRNAs, down-regulated DE-miRNAs, and up-regulated DE-mRNAs that may interact related to linoleic acid metabolism in stomach samples between suckling group and adult group of ferrets (**H**). The gold hexagons represent the up-regulated DE-lncRNAs; the aquamarine diamonds represent the down-regulated DE-miRNAs; the indianred circles represent the up-regulated DE-mRNAs.

To elucidate the interaction among DE-lncRNAs, DE-miRNAs, and DE-mRNAs between two different periods, we constructed lncRNA-miRNA-mRNA networks in red pandas and ferrets, respectively. An amount of 15 DE-lncRNAs (Figure S10A), 21 DE-miRNAs (Figure S10B), and 317 DE-mRNAs (Figure S10C) that may interact were identified in red pandas. Then, 4 up-regulated DE-lncRNAs, 4 down-regulated DE-miRNAs and 75 up-regulated DE-mRNAs that may interact were identified in adult red pandas (Figure S10D), while down-regulated DE-lncRNAs, up-regulated DE-miRNAs, and down-regulated DE-mRNAs that may interact were not identified in adult red pandas. Compared with the suckling group, the regulatory network composed of up-regulated DE-lncRNAs, down-regulated DE-miRNAs, and up-regulated DE-mRNAs in adult red pandas showed that the GO (Table S32 and Figure 6A) and KEGG (Table S33 and Figure 6B) of up-regulated DE-mRNAs were enriched in amino acid transport (GO: 0006865) category. Regulation networks of up-regulated DE-lncRNAs, down-regulated DE-miRNAs, and up-regulated DE-mRNAs that may interact between the suckling group and the adult group of red pandas were constructed (Figure 6C). Three groups of lncRNA-miRNA-mRNA regulatory relationships (MSTRG.26053.1/MSTRG.26517.1-miR-1296-*SLC38A5*, MSTRG.26516.1-miR-504-*SLC38A5*, MSTRG.26516.1-miR-346-*SLC16A10*) were identified in DE-lncRNAs, DE-miRNAs, and DE-mRNAs between different feeding periods of red pandas (Table S34 and Figure 6D), regulating the up-regulation of amino acid transporter protein genes *SLC38A5* and *SLC16A10* during the adult period, which may facilitate the efficient utilization of amino acids in bamboo. An amount of 57 DE-lncRNAs (Figure S10E), 29 DE-miRNAs (Figure S10F), and 667 DE-mRNAs (Figure S10G) that may interact were identified in ferrets. Then, 9 up-regulated DE-lncRNAs, 6 down-regulated DE-miRNAs, and 200 up-regulated DE-mRNAs that may interact were identified in adult ferrets (Figure S10H). Meanwhile, 24 down-regulated DE-lncRNAs, 10 up-regulated DE-miRNAs, and 183 down-regulated DE-mRNAs that may interact were also identified in adult ferrets (Figure S10I). Compared with the suckling group, the regulatory network composed of up-regulated DE-lncRNAs, down-regulated DE-miRNAs, and up-regulated DE-mRNAs in adult ferrets showed that the GO (Table S35 and Figure 6E) and KEGG (Table S36 and Figure 6F) of up-regulated DE-mRNAs were enriched in linoleic acid metabolism (ko00591) pathway. Regulation networks of up-regulated DE-lncRNAs, down-regulated DE-miRNAs, and up-regulated DE-mRNAs that may interact between the suckling group and the adult group of ferrets were constructed (Figure 6G). One group of lncRNA-miRNA-mRNA regulatory relationship was identified (ENSMPUT00000030417/ENSMPUT00000031746/ENSMPUT00000033161-miR-370-*ENSMPUG00000009617*/*ENSMPUG00000017129*) in DE-lncRNAs, DE-miRNAs, and DE-mRNAs of ferrets, regulating the up-regulation of linoleic acid metabolism-related genes *ENSMPUG00000009617* and *ENSMPUG00000017129* during the adult period (Table S37 and Figure 6H), which may help to reduce the risk of hyperlipidemia and other diseases easily caused by long-term meat consumption. Compared with the suckling group, the regulatory network composed of down-regulated DE-lncRNAs, up-regulated DE-miRNAs, and down-regulated DE-mRNAs in adult ferrets showed that the GO (Table S38 and Figure S11A) and KEGG (Table S39 and Figure S11B) of down-regulated DE-mRNAs were enriched in fructose and mannose metabolism (ko00051) pathway. Regulation networks of down-regulated DE-lncRNAs, up-regulated DE-miRNAs, and down-regulated DE-mRNAs that may interact between the suckling group and the adult group of ferrets were also constructed (Figure S11C).

## 4. Discussion

Red pandas are classified in the order Carnivora, but they have evolved to become specialized bamboo eaters. NcRNAs play a role in regulating gene expression, thereby affecting organ development and nutrient utilization [29,31,32,34]. The gene expression and ncRNA regulation of the digestive organs undergo adaptive changes as red pandas transition from feeding on milk to bamboo during postnatal development. This process may differ from other carnivores such as ferrets, which exhibit a close phylogenetic relationship with red pandas, but eat meat after weaning [36]. The stomach is an important digestive organ that plays a crucial role in digestion and metabolism. Our study is the first to describe the expression and ncRNA regulation of genes related to digestion and metabolism in stomach samples of red pandas and ferrets during both the suckling period and the adult period using whole transcriptome analysis. This approach helps us gain new insights into understanding the unique diet of red pandas.

### 4.1. Digestion and Metabolism of Carbohydrates

The energy-rich nutrient carbohydrate is a valuable calorie resource derived from low-fat and low-protein bamboo for adult red pandas and giant pandas, meeting their daily energy needs [64,65,66]. A previous study has demonstrated that high expression levels of genes related to carbohydrate metabolism in the liver of adult giant pandas enable them to effectively utilize carbohydrates from bamboo [9]. SLC5A4 can activate glucose sodium transporter activity and proton transmembrane transporter activity, thereby participating in carbohydrate utilization [67,68]. In comparison with the suckling period, four up-regulated DE-lncRNAs (MSTRG.12083.1, MSTRG.15071.1, MSTRG.26168.1, MSTRG.7318.1) may positively regulate the up-regulated DE-mRNA *SLC5A4* involved in carbohydrate utilization in the stomach of adult red pandas (Table S18 and Figure 5A), which may also help adult red pandas fully utilize carbohydrates from low-nutrient bamboo to meet their energy demands.

### 4.2. Digestion and Metabolism of Amino Acids and Proteins

A previous study showed that the expression levels of amino acid and protein metabolism-related genes in adult giant pandas were up-regulated, suggesting an increase in metabolic levels during the adult period [9]. We found that, similar to giant pandas, in the stomach of adult red pandas, the up-regulated DE-mRNAs (Table S3), target mRNAs of up-regulated DE-lncRNAs (Table S14), target mRNAs of down-regulated DE-miRNAs (Table S26), and up-regulated DE-mRNAs of lncRNA-miRNA-mRNA networks (Table S32) were enriched in amino acid transport (GO:0006865) category compared with the suckling period. Three groups of networks among DE-lncRNAs, DE-miRNAs, and DE-mRNAs (MSTRG.26053.1/MSTRG.26517.1-miR-1296-*SLC38A5*, MSTRG.26516.1-miR-504-*SLC38A5*, MSTRG.26516.1-miR-346-*SLC16A10*) were identified in red pandas, which may regulate the up-regulation of *SLC38A5* and *SLC16A10* involved in amino acid transport [69,70,71] during the adult period (Table S34). Meanwhile, in the stomach of adult ferrets, we observed that the target mRNAs of up-regulated DE-lncRNAs (Table S20) were significantly enriched in various amino acid degradation pathways such as valine, leucine, and isoleucine degradation (ko00280) pathway compared with the suckling period. We also identified numerous positive-correlation networks between DE-lncRNAs and DE-mRNAs (Table S23) in ferrets, which may play a role in regulating the up-regulation of *BCKDHB* and *AASS* related to amino acid degradation [72,73] during the adult period. These findings may suggest the following: firstly, ncRNAs may play a role in regulating the digestive and metabolic functions of amino acids in the stomach of adult red pandas and ferrets, leading to more efficient utilization of amino acids in food and higher levels of digestion and metabolism; secondly, the high expression levels of these genes may contribute to the efficient utilization of limited amino acids in bamboo by adult red pandas, potentially reflecting a positive adaptation to their specialized bamboo diet; thirdly, the increase in the degradation of amino acids in adult ferrets may contribute to maintaining a balance in amino acid metabolism, as adult ferrets have a higher frequency of daily intake and consume larger quantities on a daily basis [74].

The gastric acid secreted by gastric parietal cells is capable of converting pepsinogen into active pepsin, which in turn can digest dietary proteins. Studies have suggested that the secretion of gastric acid, as well as the secretion and activity of pepsin in the rat stomach, are initially extremely low at birth but significantly increase after weaning, gradually reaching adult levels [75,76,77]. Therefore, it is likely that the expression levels of genes related to pepsinogen during the adult period may be significantly higher than those during the suckling period. PGA5, PGB, and PGC all function as pepsinogens and play a crucial role in the digestion of dietary proteins [78,79,80]. Similar to rats, the pepsinogen DE-mRNAs (PGA5, PGB, and PGC) were found to be up-regulated in the adult group compared with the suckling group in red pandas (Table S7). In comparison to the suckling period, four up-regulated DE-lncRNAs (MSTRG.1791.1, MSTRG.26053.1, MSTRG.6265.1, and MSTRG.9343.1) may positively regulate the up-regulated DE-mRNA *PGA5* and *PGC* in the stomach of adult red pandas (Table S18 and Figure 5C). The elevated expression levels of pepsinogen genes in adult red pandas may also contribute to the efficient utilization of limited proteins in bamboo and higher levels of digestion and metabolism. Unlike rats and red pandas, no significant differences were observed in pepsinogen genes between the suckling and adult periods of carnivorous ferrets.

In the stomach of suckling red pandas, the up-regulated DE-mRNAs (Tables S3 and S4) were enriched in the positive regulation of protein catabolic process (GO:0045732) category and protein digestion and absorption (ko04974) pathway compared with the adult period. The pre-protein encoded by GRP can undergo hydrolysis to produce gastrin-releasing peptide, which in turn releases gastrin and stimulates the secretion of gastric acid, thereby responding to the intake of protein-rich food [81]. Compared with the adult period, *GRP* was found to be up-regulated in the stomach of suckling red pandas (Table S7). A previous study has indicated that certain trypsin genes were highly expressed in the pancreas of suckling giant pandas. This expression is believed to be beneficial for them as it allows for the extraction of high protein content from milk, which is essential for their rapid growth and development needs [9]. We hypothesized that, despite the relatively low expression levels of pepsinogen genes in suckling red pandas, other genes involved in the digestion and absorption of proteins, such as *GRP* in stomach tissues and certain trypsin genes in pancreas tissues, may play a role in efficiently utilizing the abundant protein found in milk. This efficient utilization is essential for the rapid growth and development of the red panda cubs.

### 4.3. Digestion and Metabolism of Lipids

A previous study has indicated that the expression levels of genes related to cholesterol metabolism in suckling giant pandas were up-regulated. This suggests a high metabolic rate of lipids during the suckling period [9]. Similar to the research conducted on giant pandas, this study also found that the up-regulated DE-mRNAs in the stomach of suckling red pandas were enriched in response to fatty acid (GO:0070542) category, fatty acid binding (GO:0005504) category, and cholesterol metabolism (ko04979) pathway. Cholecystokinin can bind to the cholecystokinin receptor encoded by *CCKAR* in order to stimulate the contraction of gastric smooth muscle. This response is triggered by the intake of food containing fat and protein [81,82]. We observed that, in comparison to adulthood, the up-regulation of *CCKAR* expression level in the stomach of suckling red pandas (Table S7 and Figure 4G) may be associated with the high lipid and protein content in milk, as well as the substantial amount of fat and protein requiring digestion and absorption. Unlike red pandas, the up-regulated DE-mRNAs in the stomach of adult ferrets were found to be enriched in cholesterol transfer activity (GO:012020) category and linoleic acid metabolism (ko00591) pathway. The increased linoleic acid metabolism, which promotes cholesterol decomposition [83], may prove beneficial in reducing the risk of hyperlipidemia and arteriosclerosis that can easily occur as a result of long-term meat consumption in adult ferrets. The up-regulated DE-lncRNAs in adult ferrets were found to have target genes enriched in lipid transporter activity (GO:0005319) category and fatty acid degradation (ko00071) pathway. The positive regulation of these target genes by DE-lncRNAs may assist ferrets in effectively utilizing meat fat. Additionally, the up-regulation of DE-mRNAs *ENSMPUG00000009617* and *ENSMPUG00000017129*, which are related to linoleic acid metabolism during the adult period, is regulated by DE-lncRNAs and DE-miRNAs (Table S37). This regulatory mechanism may contribute to reducing the risk of diseases that can be easily caused by long-term meat consumption.

The main discussion results were as follows: (1) in terms of carbohydrates, up-regulated gene related to carbohydrate transport regulated by lncRNAs may help adult red pandas fully utilize carbohydrates of low-energy bamboo to meet their energy requirements; (2) regarding amino acids, up-regulated genes related to amino acid transport regulated by lncRNA-miRNA-mRNA networks may enable efficient utilization of limited bamboo amino acids in adult red pandas, while up-regulated genes related to amino acid degradation regulated by lncRNAs may help maintain a balance in amino acid metabolism due to larger daily intakes in adult ferrets; (3) with respect to proteins, we speculated that certain digestion-related genes in stomach tissue and trypsin genes in pancreas tissue, rather than pepsinogen genes, play more critical roles in utilizing the abundant proteins in milk required for the rapid growth and development of suckling red pandas; (4) lastly, as for lipids, some up-regulated genes related to lipid digestion may contribute to the utilization of high-fat milk for the rapid growth and development of suckling red pandas, while up-regulated genes related to linoleic acid metabolism regulated by lncRNA-miRNA-mRNA networks may promote cholesterol decomposition to reduce health risks for adult ferrets.

## 5. Conclusions and Limitations

This study primarily investigated the changes in expression of digestion- and metabolism-related genes, as well as the regulatory roles of ncRNAs (lncRNAs, miRNAs), from the suckling period to the adult bamboo-eating period in red pandas. This was achieved using data obtained from stomach tissue samples of red pandas and ferrets during both the suckling and adult periods. The main findings revealed that, under the regulation of ncRNAs, certain up-regulated genes related to lipid digestion in suckling red pandas may facilitate their utilization of the high fat content in milk for rapid growth and development. Additionally, some up-regulated genes associated with carbohydrate and amino acid transport in adult red pandas may indicate their heightened metabolic levels and help them utilize the limited energy and nutrients from bamboo for daily activities. Our study provides evidence of gene expression adaptation and ncRNA regulation for the specific dietary shift and nutrient utilization in red pandas.

There are two major limitations in this study that could be addressed in future research: first, our study lacks sufficient sequential sampling across development due to the difficulty in obtaining precious samples of red pandas; second, our study verified the expression levels of some digestion- and metabolism-related genes in red pandas, while lacking experimental verification of the regulatory effects of ncRNAs on corresponding gene expression levels due to the difficulty in conducting experimental verification on endangered protected animal red pandas.

## Data Availability

The whole transcriptome sequencing data of red pandas and ferrets in this study have been submitted to the NCBI Sequence Read Archive (accession number PRJNA1098497).

## Supplementary Materials

The following supporting information can be downloaded at: https://www.mdpi.com/article/10.3390/ani14121795/s1, Figure S1: Characteristics of lncRNAs and miRNAs in red pandas and ferrets; Figure S2: Enrichment analyses for DE-mRNAs between two different feeding periods in stomach samples of red pandas; Figure S3: Enrichment analyses for DE-mRNAs between two different feeding periods in stomach samples of ferrets; Figure S4: The expression trends of the up-regulated DE-mRNAs associated with linoleic acid metabolism (A,B) and amino acid degradation (C,D) in the stomach samples of the adult group compared with the suckling group in ferrets; Figure S5: Enrichment analyses for target mRNAs of DE-lncRNAs between two different feeding periods in stomach samples of red pandas; Figure S6: Enrichment analyses for target mRNAs of DE-lncRNAs between two different feeding periods in stomach samples of ferrets; Figure S7: The interaction networks of DE-lncRNAs and DE-mRNAs associated with linoleic acid metabolism (A) and amino acid degradation (B) in stomach samples between suckling group and adult group of ferrets; Figure S8: Enrichment analyses for target mRNAs of DE-miRNAs between two different feeding periods in stomach samples of red pandas; Figure S9: Enrichment analyses for target mRNAs of DE-miRNAs between two different feeding periods in stomach samples of ferrets; Figure S10: Identification of DE-lncRNAs, DE-miRNAs, and DE-mRNAs that may interact in stomach samples between suckling group and adult group of red pandas and ferrets; Figure S11: Enrichment analyses and regulation network of DE-lncRNAs, DE-miRNAs, and DE-mRNAs that may interact in stomach samples between suckling group and adult group of ferrets; Table S1: The descriptions of the 12 strand-specific RNA-seq libraries for two mammals used in this study; Table S2: The descriptions of the 12 sRNA-seq libraries for two mammals used in this study; Table S3: Significantly enriched GO categories for up-regulated DE-mRNAs in stomach samples of adult group compared with the suckling group in red pandas. The significantly enriched top 60 categories were selected for display; Table S4: Significantly enriched KEGG categories for up-regulated DE-mRNAs in stomach samples of adult group compared with the suckling group in red pandas; Table S5: Significantly enriched GO categories for down-regulated DE-mRNAs in stomach samples of adult group compared with the suckling group in red pandas. The significantly enriched top 60 categories were selected for display; Table S6: Significantly enriched KEGG categories for down-regulated DE-mRNAs in stomach samples of adult group compared with the suckling group in red pandas; Table S7: DE-mRNAs related to digestion and metabolism in stomach samples of adult group compared with the suckling group in red pandas; Table S8: Primer sequences of 9 DE-mRNAs between two different feeding periods in red pandas for real-time PCR; Table S9: Significantly enriched GO categories for up-regulated DE-mRNAs in stomach samples of adult group compared with the suckling group in ferrets. The significantly enriched top 60 categories were selected for display; Table S10: Significantly enriched KEGG categories for up-regulated DE-mRNAs in stomach samples of adult group compared with the suckling group in ferrets; Table S11: Significantly enriched GO categories for down-regulated DE-mRNAs in stomach samples of adult group compared with the suckling group in ferrets. The significantly enriched top 60 categories were selected for display; Table S12: Significantly enriched KEGG categories for down-regulated DE-mRNAs in stomach samples of adult group compared with the suckling group in ferrets; Table S13: DE-mRNAs related to digestion and metabolism in stomach samples of adult group compared with the suckling group in ferrets; Table S14: Significantly enriched GO categories for target mRNAs of up-regulated DE-lncRNAs in stomach samples of adult group compared with the suckling group in red pandas. The significantly enriched top 60 categories were selected for display; Table S15: Significantly enriched KEGG categories for target mRNAs of up-regulated DE-lncRNAs in stomach samples of adult group compared with the suckling group in red pandas; Table S16: Significantly enriched GO categories for target mRNAs of down-regulated DE-lncRNAs in stomach samples of adult group compared with the suckling group in red pandas. The significantly enriched top 60 categories were selected for display; Table S17: Significantly enriched KEGG categories for target mRNAs of down-regulated DE-lncRNAs in stomach samples of adult group compared with the suckling group in red pandas; Table S18: target mRNAs of DE-lncRNAs related to digestion and metabolism in stomach samples of adult group compared with the suckling group in red pandas; Table S19: Significantly enriched GO categories for target mRNAs of up-regulated DE-lncRNAs in stomach samples of adult group compared with the suckling group in ferrets. The significantly enriched top 60 categories were selected for display; Table S20: Significantly enriched KEGG categories for target mRNAs of up-regulated DE-lncRNAs in stomach samples of adult group compared with the suckling group in ferrets; Table S21: Significantly enriched GO categories for target mRNAs of down-regulated DE-lncRNAs in stomach samples of adult group compared with the suckling group in ferrets. The significantly enriched top 60 categories were selected for display; Table S22: Significantly enriched KEGG categories for target mRNAs of down-regulated DE-lncRNAs in stomach samples of adult group compared with the suckling group in ferrets; Table S23: target mRNAs of DE-lncRNAs related to digestion and metabolism in stomach samples of adult group compared with the suckling group in ferrets; Table S24: Significantly enriched GO categories for target mRNAs of up-regulated DE-miRNAs in stomach samples of adult group compared with the suckling group in red pandas. The significantly enriched top 60 categories were selected for display; Table S25: Significantly enriched KEGG categories for target mRNAs of up-regulated DE-miRNAs in stomach samples of adult group compared with the suckling group in red pandas; Table S26: Significantly enriched GO categories for target mRNAs of down-regulated DE-miRNAs in stomach samples of adult group compared with the suckling group in red pandas. The significantly enriched top 60 categories were selected for display; Table S27: Significantly enriched KEGG categories for target mRNAs of down-regulated DE-miRNAs in stomach samples of adult group compared with the suckling group in red pandas; Table S28: Significantly enriched GO categories for target mRNAs of up-regulated DE-miRNAs in stomach samples of adult group compared with the suckling group in ferrets. The significantly enriched top 60 categories were selected for display; Table S29: Significantly enriched KEGG categories for target mRNAs of up-regulated DE-miRNAs in stomach samples of adult group compared with the suckling group in ferrets; Table S30: Significantly enriched GO categories for target mRNAs of down-regulated DE-miRNAs in stomach samples of adult group compared with the suckling group in ferrets. The significantly enriched top 60 categories were selected for display; Table S31: Significantly enriched KEGG categories for target mRNAs of down-regulated DE-miRNAs in stomach samples of adult group compared with the suckling group in ferrets; Table S32: Significantly enriched GO categories for DE-mRNAs of regulatory network composed of up-regulated DE-lncRNAs, down-regulated DE-miRNAs, and up-regulated DE-mRNAs in stomach samples of adult group compared with the suckling group in red pandas. The significantly enriched top 60 categories were selected for display; Table S33: Significantly enriched KEGG pathways for DE-mRNAs of regulatory network composed of up-regulated DE-lncRNAs, down-regulated DE-miRNAs, and up-regulated DE-mRNAs in stomach samples of adult group compared with the suckling group in red pandas; Table S34: Regulation networks of up-regulated DE-lncRNAs, down-regulated DE-miRNAs, and up-regulated DE-mRNAs that may interact related to digestion and metabolism in stomach samples of adult group compared with the suckling group in red pandas; Table S35: Significantly enriched GO categories for DE-mRNAs of regulatory network composed of up-regulated DE-lncRNAs, down-regulated DE-miRNAs, and up-regulated DE-mRNAs in stomach samples of adult group compared with the suckling group in ferrets. The significantly enriched top 60 categories were selected for display; Table S36: Significantly enriched KEGG pathways for DE-mRNAs of regulatory network composed of up-regulated DE-lncRNAs, down-regulated DE-miRNAs, and up-regulated DE-mRNAs in stomach samples of adult group compared with the suckling group in ferrets; Table S37: Regulation networks of up-regulated DE-lncRNAs, down-regulated DE-miRNAs, and up-regulated DE-mRNAs that may interact related to digestion and metabolism in stomach samples of adult group compared with the suckling group in ferrets; Table S38: Significantly enriched GO categories for DE-mRNAs of regulatory network composed of down-regulated DE-lncRNAs, up-regulated DE-miRNAs, and down-regulated DE-mRNAs in stomach samples of adult group compared with the suckling group in ferrets. The significantly enriched top 60 categories were selected for display; Table S39: Significantly enriched KEGG pathways for DE-mRNAs of regulatory network composed of down-regulated DE-lncRNAs, up-regulated DE-miRNAs, and down-regulated DE-mRNAs in stomach samples of adult group compared with the suckling group in ferrets.
