# Supplementary material for: Adaptive Expression and ncRNA Regulation of Genes Related to Digestion and Metabolism in Stomach of Red Pandas during Suckling and Adult Periods
Source: Animals (Basel). 2024 Jun 15;14(12):1795. doi: 10.3390/ani14121795 (PMC11200446; doi:10.3390/ani14121795)
Supplement: Supplementary file 1 [file animals-14-01795-s001.zip › Supplementary Figures.pdf]

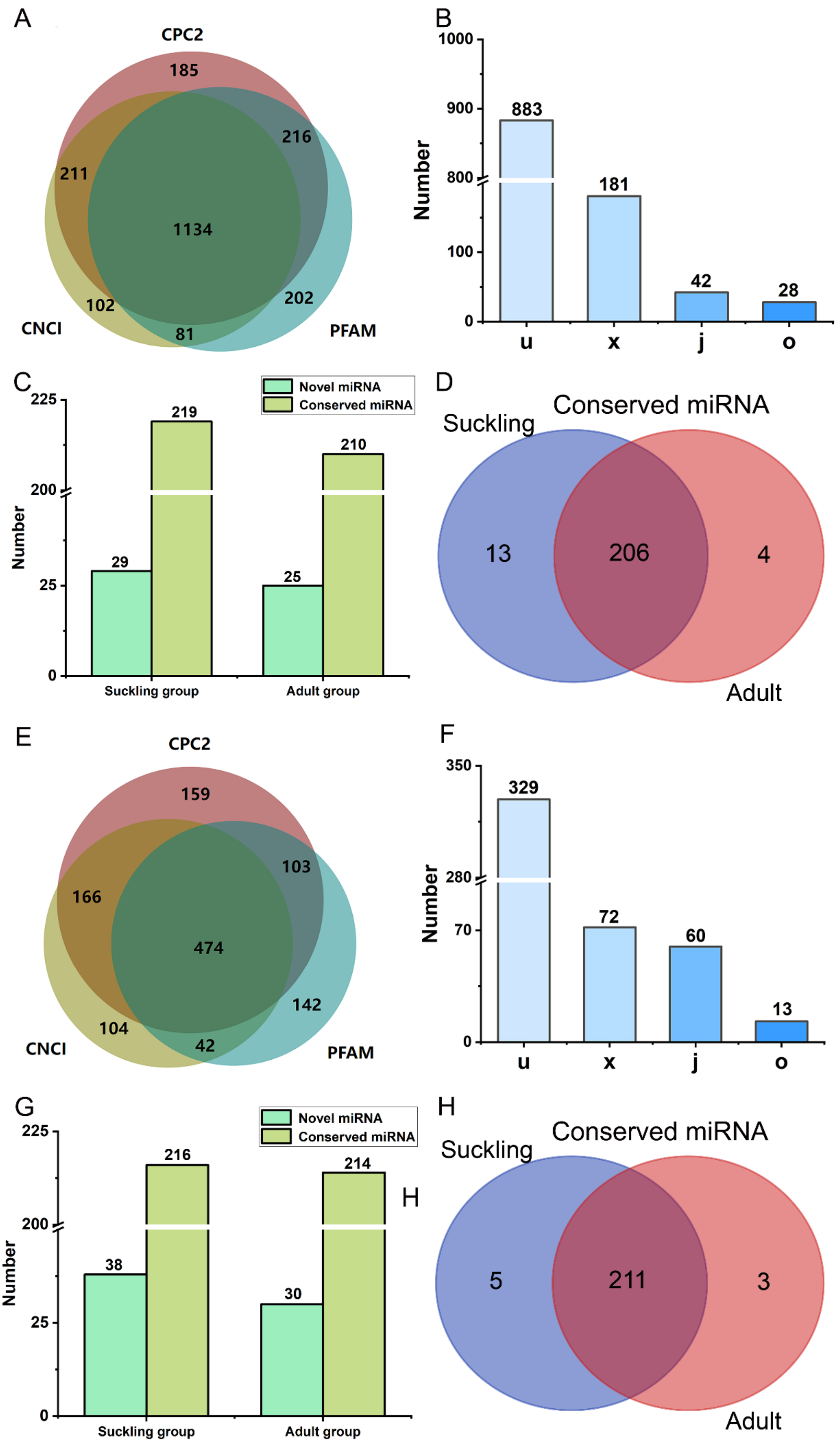
A
CPC2
185
216
211
1134
102
202
81
CNCI
PFAM
B
Number
1000
900
800
100
0
883
181
42
28
u
x
j
o
C
225
200
25
0
Number
Novel miRNA
Conserved miRNA
219
210
29
25
Suckling group
Adult group
D
Conserved miRNA
Suckling
13
206
4
Adult
E
CPC2
159
103
166
474
104
142
42
CNCI
PFAM
F
350
280
70
0
Number
329
72
60
13
u
x
j
o
G
225
200
25
0
Number
Novel miRNA
Conserved miRNA
216
214
38
30
Suckling group
Adult group
H
H
Conserved miRNA
Suckling
5
211
3
Adult

Figure S1 Characteristics of lncRNAs and miRNAs in red pandas and ferrets. Venn diagram showing the results from CNCI, CPC2 and PFAMSCAN, taking the common intersection as the newly assembled lncRNAs in red panda stomach (A). Classification of new lncRNAs from red panda stomach (B). The number of novel and known miRNAs in the stomach samples of the suckling group and the adult group of red pandas (C). The number of known miRNAs in the stomach samples shared by the suckling group and the adult group of red pandas is showed by the intersection of Venn diagram (D). Venn diagram showing the results from CNCI, CPC2 and PFAMSCAN, taking the common intersection as the newly assembled lncRNAs in ferret stomach (E). Classification of new lncRNAs from ferret stomach (F). The number of novel and known miRNAs in the stomach samples of the suckling group and the adult group of ferrets (G). The number of known miRNAs in the stomach samples shared by the suckling group and the adult group of ferrets is showed by the intersection of Venn diagram (H).

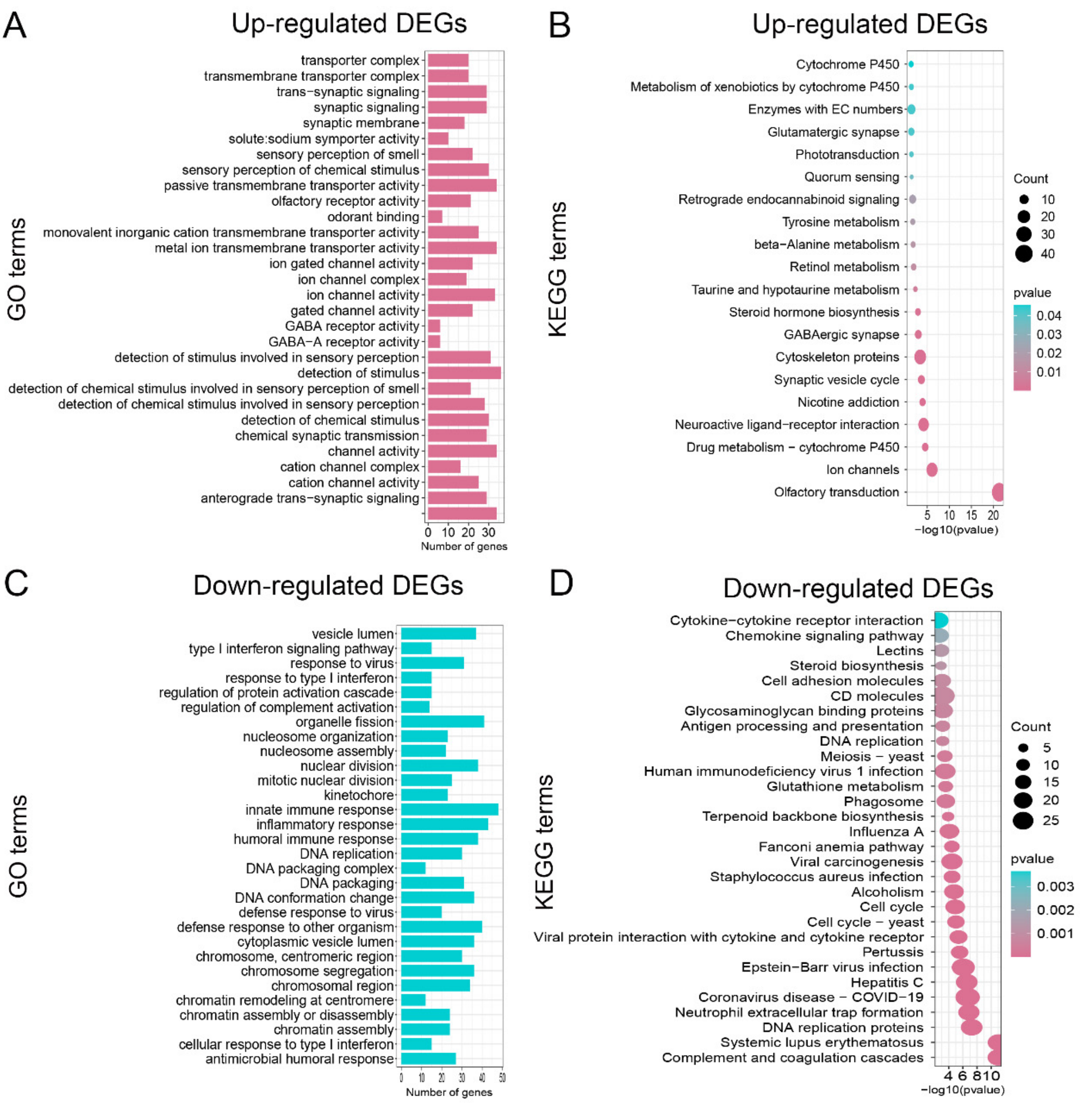

Figure S2 Enrichment analyses for DE-mRNAs between two different feeding periods in stomach samples of red pandas. Significantly enriched GO (A) and KEGG (B) categories for up-regulated DE-mRNAs in stomach samples of adult group compared with the suckling group in red pandas. Significantly enriched GO (C) and KEGG (D) categories for down-regulated DE-mRNAs in stomach samples of adult group compared with the suckling group in red pandas. The significantly enriched top 30 categories were selected for display.

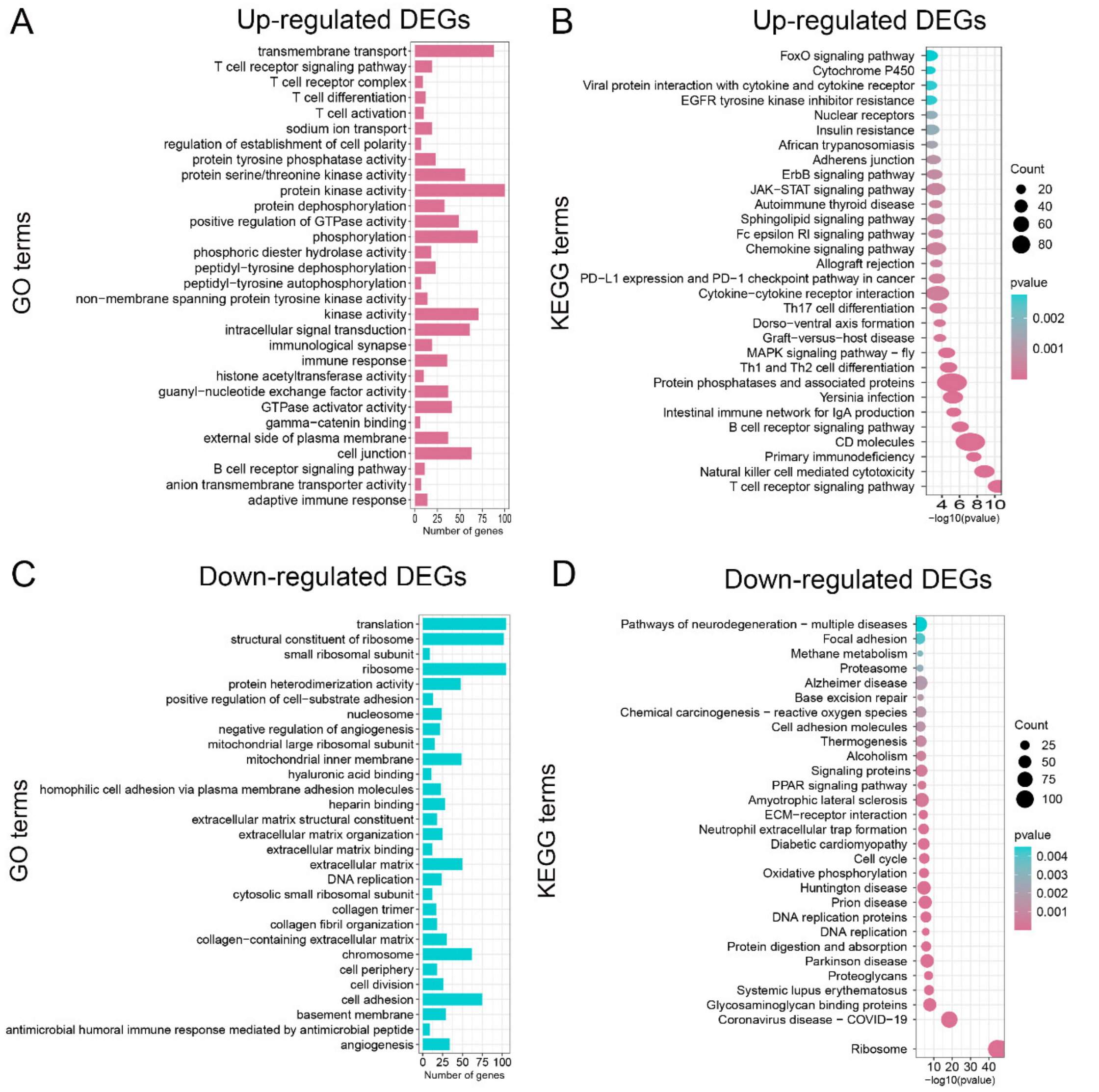

Figure S3 Enrichment analyses for DE-mRNAs between two different feeding periods in stomach samples of ferrets. Significantly enriched GO (A) and KEGG (B) categories for up-regulated DE-mRNAs in stomach samples of adult group compared with the suckling group in ferrets. Significantly enriched GO (C) and KEGG (D) categories for down-regulated DE-mRNAs in stomach samples of adult group compared with the suckling group in ferrets. The significantly enriched top 30 categories were selected for display.

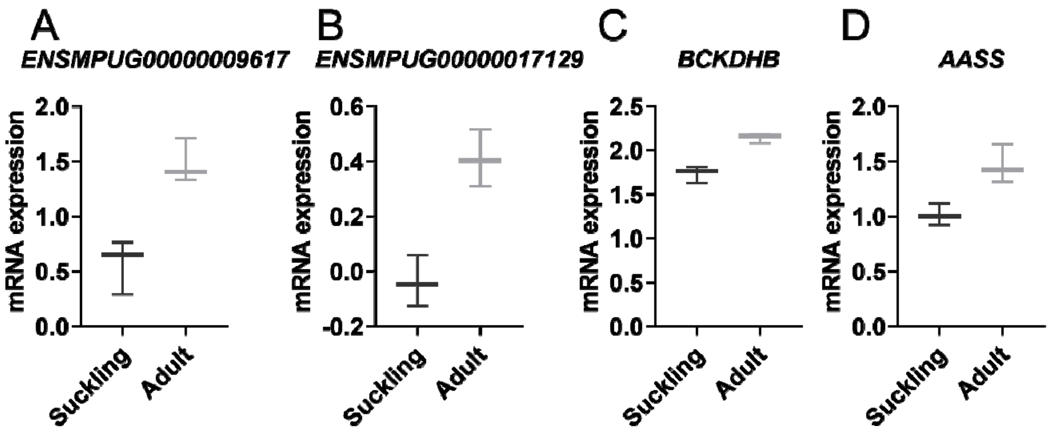

Figure S4 The expression trends of the up-regulated DE-mRNAs associated with linoleic acid metabolism (A, B) and amino acid degradation (C, D) in the stomach samples of the adult group compared with the suckling group in ferrets. The Y-axis represents normalized and log-transformed mRNA expression level. The boxplot edges indicate the 25th and 75th percentiles.

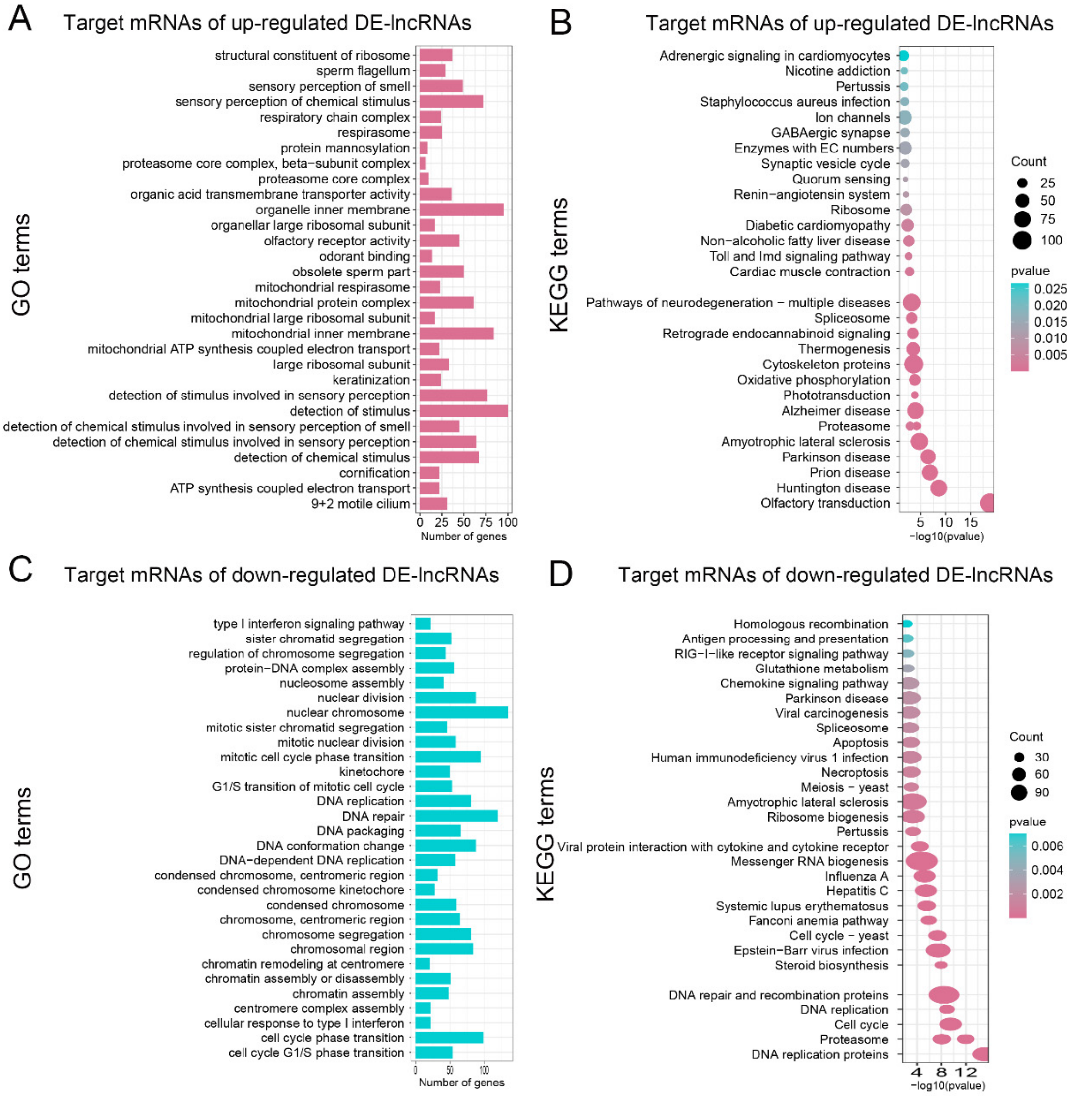

Figure S5 Enrichment analyses for target mRNAs of DE-lncRNAs between two different feeding periods in stomach samples of red pandas. Significantly enriched GO (A) and KEGG (B) categories for target mRNAs of up-regulated DE-lncRNAs in stomach samples of adult group compared with the suckling group in red pandas. Significantly enriched GO (C) and KEGG (D) categories for target mRNAs of down-regulated DE-lncRNAs in stomach samples of adult group compared with the suckling group in red pandas. The significantly enriched top 30 categories were selected for display.

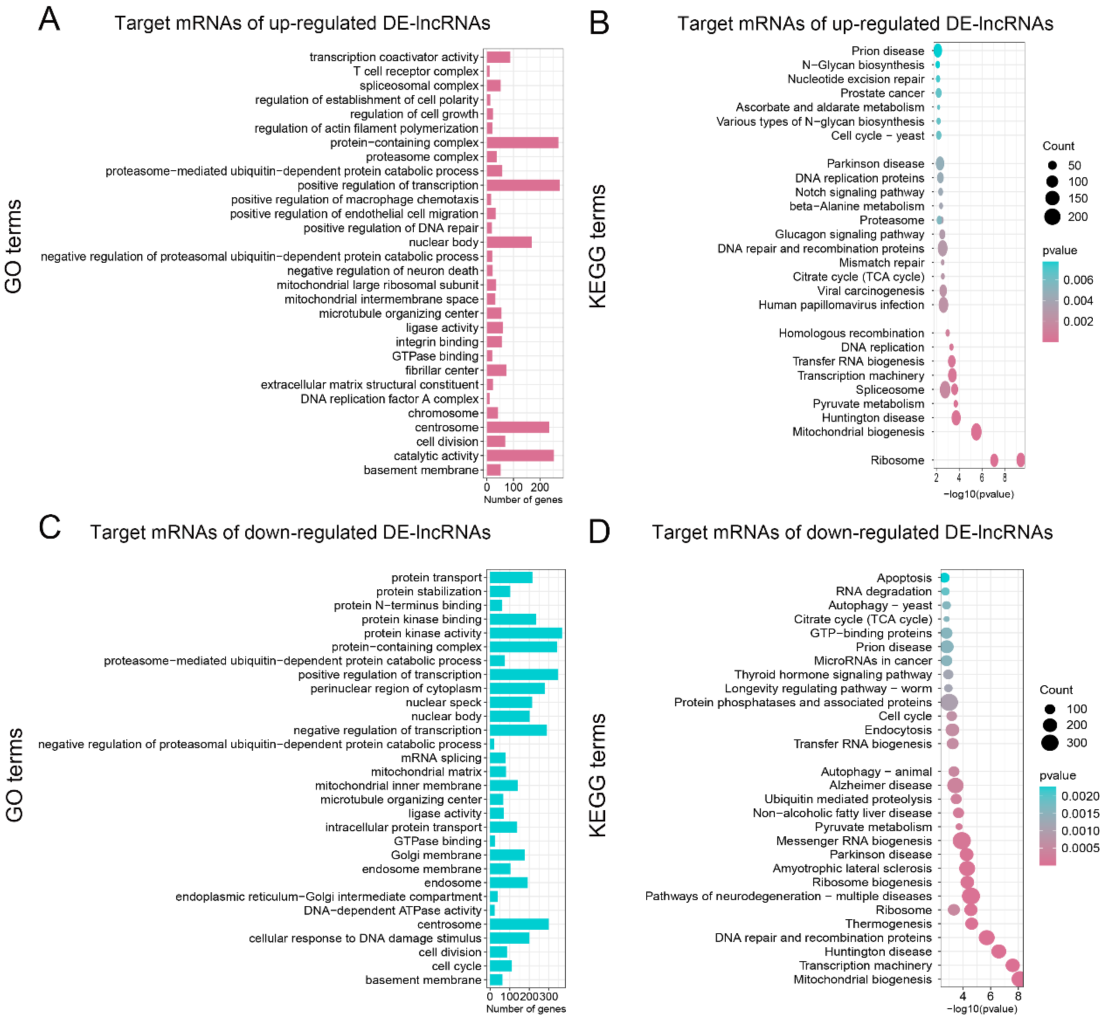

Figure S6 Enrichment analyses for target mRNAs of DE-lncRNAs between two different feeding periods in stomach samples of ferrets. Significantly enriched GO (A) and KEGG (B) categories for target mRNAs of up-regulated DE-lncRNAs in stomach samples of adult group compared with the suckling group in ferrets. Significantly enriched GO (C) and KEGG (D) categories for target mRNAs of down-regulated DE-lncRNAs in stomach samples of adult group compared with the suckling group in ferrets. The significantly enriched top 30 categories were selected for display.

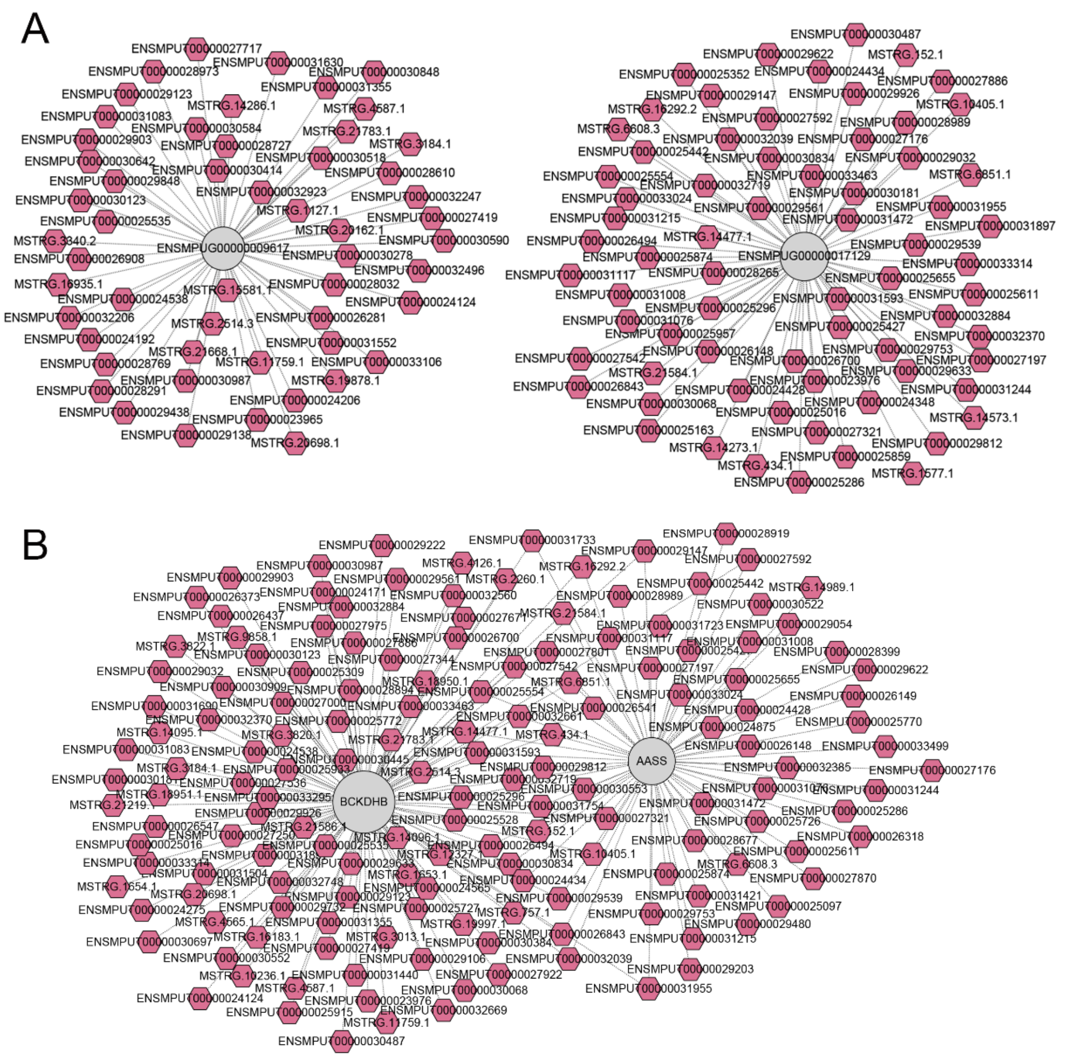

Figure S7 The interaction networks of DE-lncRNAs and DE-mRNAs associated with linoleic acid metabolism (A) and amino acid degradation (B) in stomach samples between suckling group and adult group of ferrets. The palevioletred hexagons represent the up-regulated DE-lncRNAs of adult group; the circles represent the target mRNAs of DE-lncRNAs.

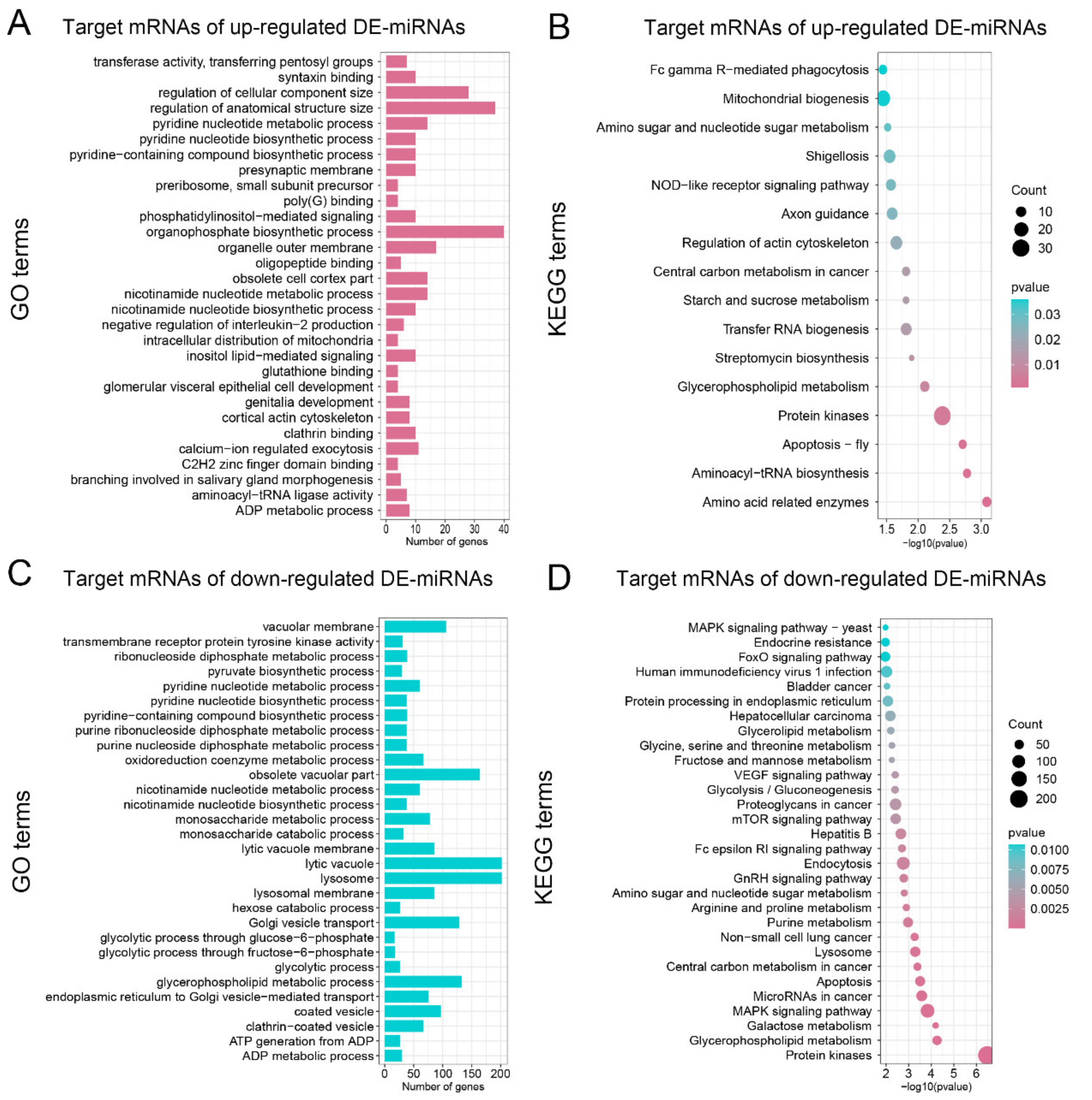

Figure S8 Enrichment analyses for target mRNAs of DE-miRNAs between two different feeding periods in stomach samples of red pandas. Significantly enriched GO (A) and KEGG (B) categories for target mRNAs of up-regulated DE-miRNAs in stomach samples of adult group compared with the suckling group in red pandas. Significantly enriched GO (C) and KEGG (D) categories for target mRNAs of down-regulated DE-miRNAs in stomach samples of adult group compared with the suckling group in red pandas. The significantly enriched top 30 categories were selected for display.

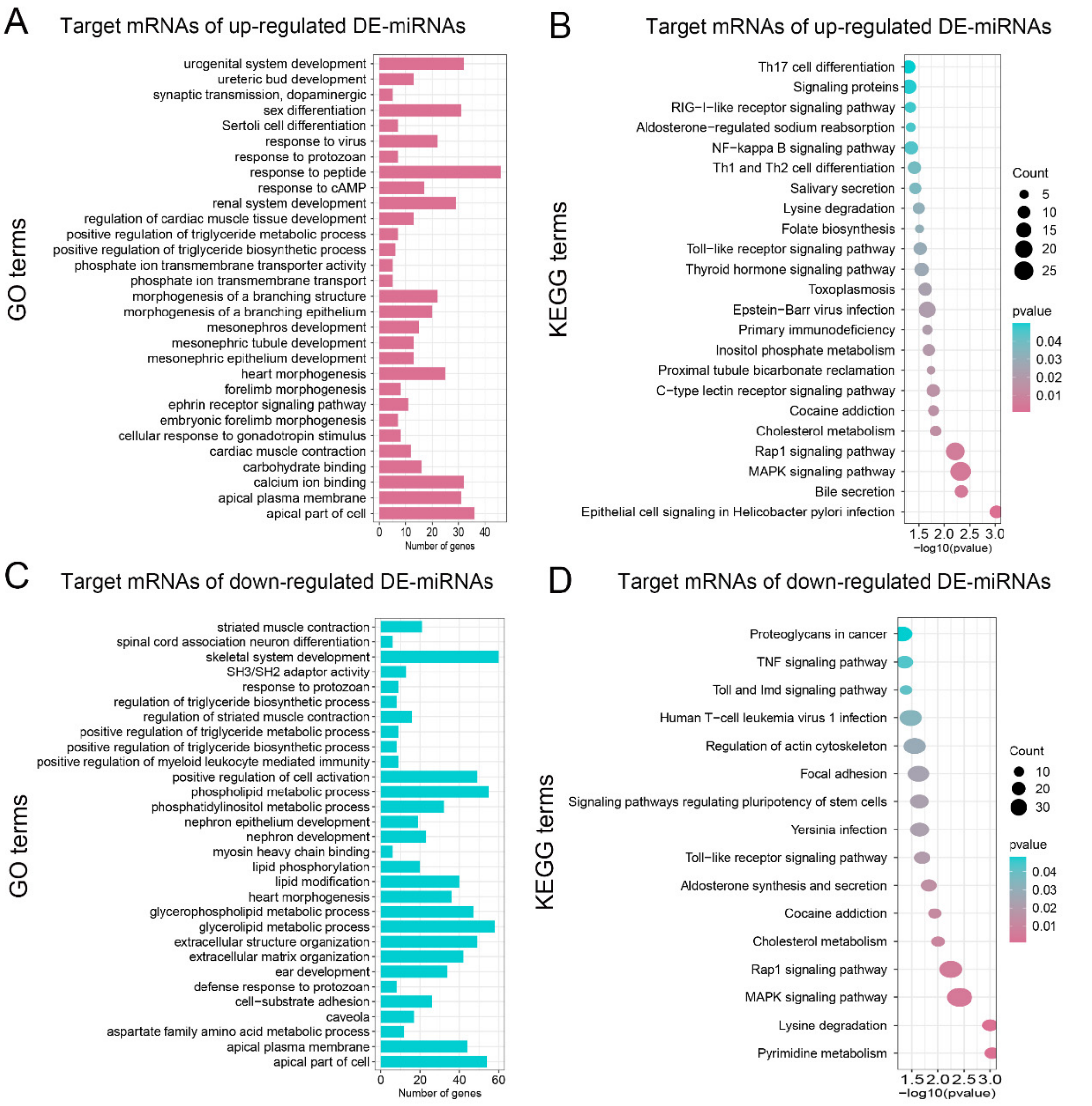

Figure S9 Enrichment analyses for target mRNAs of DE-miRNAs between two different feeding periods in stomach samples of ferrets. Significantly enriched GO (A) and KEGG (B) categories for target mRNAs of up-regulated DE-miRNAs in stomach samples of adult group compared with the suckling group in ferrets. Significantly enriched GO (C) and KEGG (D) categories for target mRNAs of down-regulated DE-miRNAs in stomach samples of adult group compared with the suckling group in ferrets. The significantly enriched top 30 categories were selected for display.

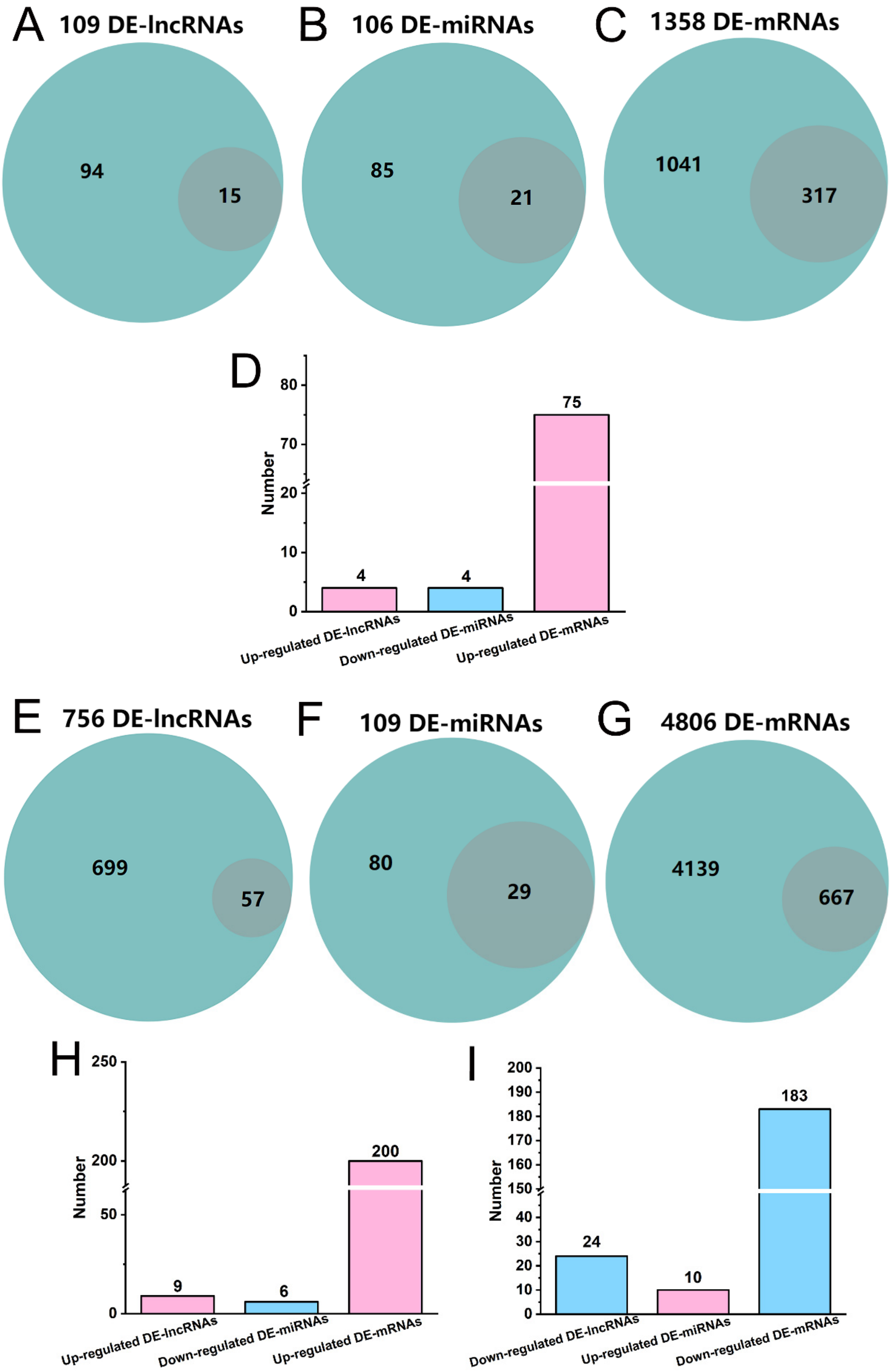

A
109 DE-lncRNAs
94
15
B
106 DE-miRNAs
85
21
C
1358 DE-mRNAs
1041
317
D
Number
80
70
20
10
0
75
4
4
Up-regulated DE-lncRNAs
Down-regulated DE-miRNAs
Up-regulated DE-mRNAs
E
756 DE-lncRNAs
699
57
F
109 DE-miRNAs
80
29
G
4806 DE-mRNAs
4139
667
H
Number
250
200
50
0
9
6
200
Up-regulated DE-lncRNAs
Down-regulated DE-miRNAs
Up-regulated DE-mRNAs
I
Number
200
190
180
170
160
150
40
30
20
10
0
24
10
183
Down-regulated DE-lncRNAs
Up-regulated DE-miRNAs
Down-regulated DE-mRNAs

Figure S10 Identification of DE-lncRNAs, DE-miRNAs and DE-mRNAs that may interact in stomach samples between suckling group and adult group of red pandas and ferrets. Venn diagrams of DE-lncRNAs (A), DE-miRNAs (B) and DE-mRNAs (C) that may interact in stomach samples between suckling group and adult group of red pandas. The numbers in the green circles represent the numbers of DE-lncRNAs (A), DE-miRNAs (B) and DE-mRNAs (C). The numbers in the gray circles represent the numbers of DE-lncRNAs (A), DE-miRNAs (B) and DE-mRNAs (C) that may interact as predicted by miRanda and RNAhybrid. The numbers of up-regulated DE-lncRNAs, down-regulated DE-miRNAs and up-regulated DE-mRNAs that may interact in stomach samples between suckling group and adult group of red pandas (D). Venn diagrams of DE-lncRNAs (E), DE-miRNAs (F) and DE-mRNAs (G) that may interact in stomach samples between suckling group and adult group of ferrets. The numbers in the green circles represent the numbers of DE-lncRNAs (E), DE-miRNAs (F) and DE-mRNAs (G). The numbers in the gray circles represent the numbers of DE-lncRNAs (E), DE-miRNAs (F) and DE-mRNAs (G) that may interact as predicted by miRanda and RNAhybrid. The numbers of up-regulated DE-lncRNAs, down-regulated DE-miRNAs and up-regulated DE-mRNAs that may interact (H), and the numbers of down-regulated DE-lncRNAs, up-regulated DE-miRNAs and down-regulated DE-mRNAs that may interact (I) in stomach samples between suckling group and adult group of ferrets.

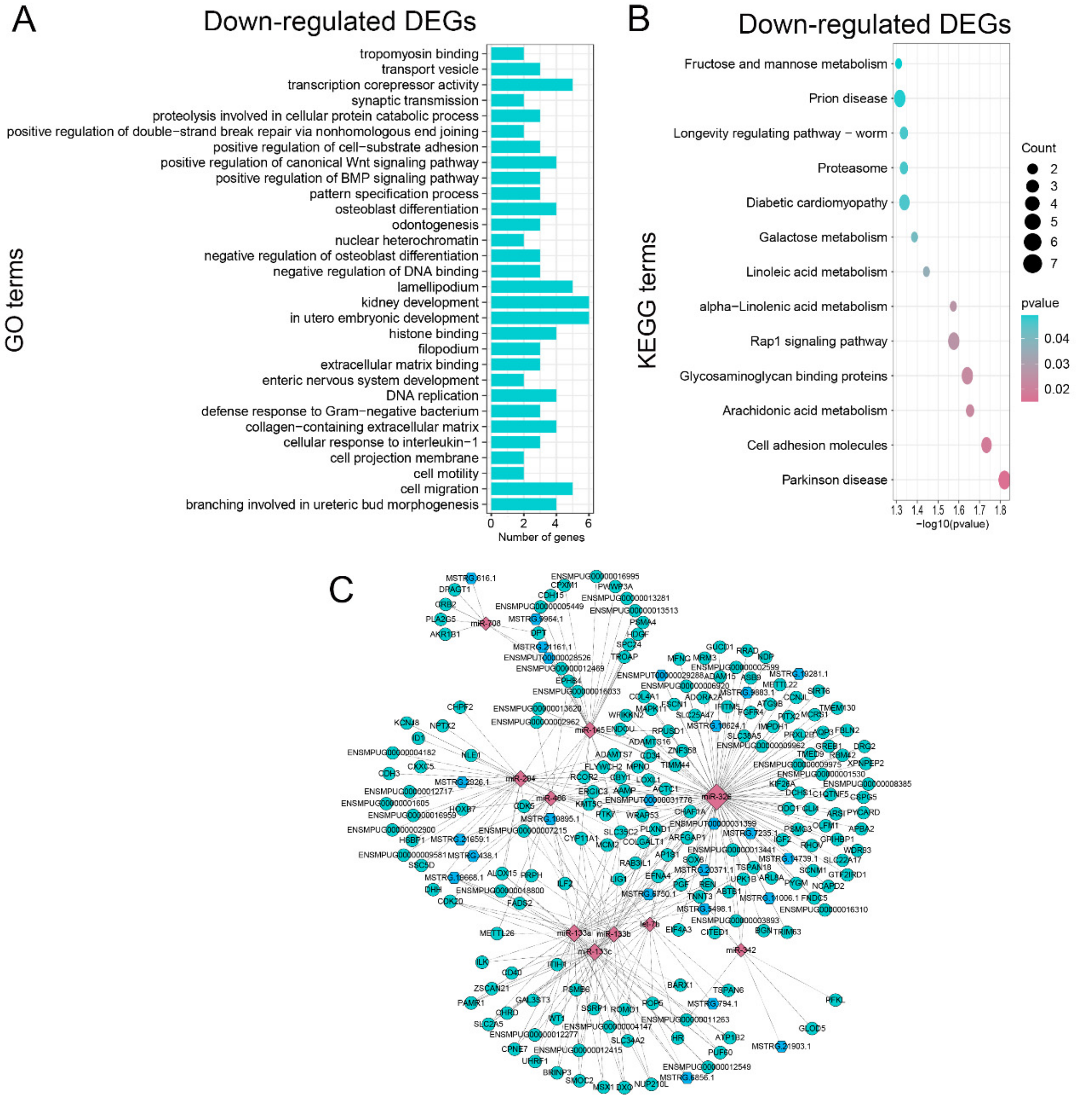

Figure S11 Enrichment analyses and regulation network of DE-lncRNAs, DE-miRNAs and DE-mRNAs that may interact in stomach samples between suckling group and adult group of ferrets. Significantly enriched GO categories (A) and KEGG pathways (B) for DE-mRNAs of regulatory network composed of down-regulated DE-lncRNAs, up-regulated DE-miRNAs and down-regulated DE-mRNAs in stomach samples of adult group compared with the suckling group in ferrets. The significantly enriched top 30 categories were selected for display. Regulation network of down-regulated DE-lncRNAs, up-regulated DE-miRNAs and down-regulated DE-mRNAs that may interact in stomach samples between suckling group and adult group of ferrets (C). The deepskyblue hexagons represent the down-regulated DE-lncRNAs; the palevioletred diamonds represent the up-regulated DE-miRNAs; the indianred circles represent the down-regulated DE-mRNAs.
